# Prevalence of suicidal behavior in patients with chronic pain: a systematic review and meta-analysis of observational studies

**DOI:** 10.3389/fpsyg.2023.1217299

**Published:** 2023-09-29

**Authors:** Chan-Young Kwon, Boram Lee

**Affiliations:** ^1^Department of Oriental Neuropsychiatry, Dong-Eui University College of Korean Medicine, Busan, Republic of Korea; ^2^KM Science Research Division, Korea Institute of Oriental Medicine, Daejeon, Republic of Korea

**Keywords:** chronic pain, suicide, suicidal behavior, suicidal ideation, suicide attempt, meaning in life, demoralization

## Abstract

**Objective:**

Chronic pain is a leading cause of disability, severely impairing an individual’s daily activity and quality of life. In addition, this condition may contribute to suicidal thoughts by leading to neuropsychological impairments, a perceived lack of meaning in life, and pain-related catastrophizing. This systematic review aimed to comprehensively investigate the prevalence and associated factors of suicidal behaviors (SBs) including suicidal ideation (SI) and suicide attempt (SA) or its complete, in individuals with chronic pain.

**Methods:**

Five electronic databases were searched up to October 4, 2022. Only observational studies investigating the prevalence of SB in individuals with chronic pain were included. The methodological quality of the included studies was assessed using the Strengthening the Reporting of Observational Studies in Epidemiology (STROBE) statement. A meta-analysis was conducted to quantify the prevalence of SB in the population, and the command “Metaprop” was used in STATA/MP 16. In addition, factors explaining the association between chronic pain and SB identified through regression analysis were investigated.

**Results:**

A total of 19 studies were included in this review (*N* = 3,312,343). The pooled lifetime prevalence of SI and SA was 28.90% (95% confidence interval, 17.95 to 41.26%) and 10.83% (5.72 to 17.30%), respectively, in a mixed sample comprising various chronic pain conditions. Importantly, the pooled prevalence of past 2-week SI was as high as 25.87% (18.09 to 34.50%). The methodological quality of the included studies was not optimal, and studies using validated SB assessment tools were lacking. Potential protective factors against SB in this population included pain coping and self-efficacy, older age, certain race/ethnicity groups, and marriage.

**Conclusion:**

This systematic review and meta-analysis demonstrated the high prevalence of SB in individuals with chronic pain. Specifically, around 1 in 4 individuals with chronic pain had SI within the last 2 weeks. However, there was considerable heterogeneity in the pooled prevalence of SB in this population.

## 1. Introduction

In general, chronic pain refers to persistent or recurrent pain that lasts for longer than 3 months (Treede et al., 2015). Chronic pain can be attributed to various causes, including primary pain, cancer pain, postoperative pain, posttraumatic pain, neuropathic pain, headache, orofacial pain, visceral pain, and musculoskeletal pain (Treede et al., 2015). According to a recent meta-analysis, the overall pooled prevalence of chronic pain in young adults was 11.6%, which has been recognized as a common public health problem (Murray et al., 2022). The prevalence of chronic pain is higher in the elderly population, and the prevalence of neuropathic pain, which is an important cause of chronic pain in the population, is estimated to be up to 50% depending on the study (Domenichiello and Ramsden, 2019). Chronic pain poses a huge socio-economic burden as it is associated with lost productivity, absenteeism, early retirement costs, and the use of healthcare services (Breivik et al., 2013). For example, back pain and neck pain, common causes of chronic pain, were health conditions with the third highest cost in the United States from 1996 to 2013, with an estimated cost of $87.6 billion (Dieleman et al., 2016).

Chronic pain is a leading cause of disability, impairing an individual’s daily activity and quality of life (Hooten, 2016). Accordingly, chronic pain often has a bidirectional association with poor mental health status (Hooten, 2016). Chronic pain can lead to depression and is thought to be associated with suicidality by causing hopelessness and promoting the desire to escape through death (Wilson et al., 2013; Hooley et al., 2014). Chronic pain also has the potential to affect suicidal tendencies in relation to changes in brain circuits that mediate reward and anti-reward pathways (Elman et al., 2013). In a biopsychosocial framework, chronic pain can contribute to suicidal thoughts by leading to neuropsychological impairments, a perceived lack of meaning in life (MiL), and pain-related catastrophizing (Edwards et al., 2006; Costanza et al., 2021). Importantly, loss of MiL is one of the subcomponents of demoralization that is the major reason individual seek psychiatric treatment, and associated with poor outcomes in physical and psychiatric illness as well as suicidal ideation (SI) (Clarke and Kissane, 2002). According to a large sample analysis in the United States, from 2003 to 2014, 8.8% of approximately 120,000 suicide decedents had chronic pain, and the proportion appeared to increase over time (Petrosky et al., 2018). In addition, according to a national representative sample analysis in the United States, chronic headache and myalgia were significantly associated with SI in adolescents (odds ratio = 1.2 to 1.3) after controlling for depressive symptoms (van Tilburg et al., 2011).

To date, various interventions, including psychotropic medications, have been investigated for suicide and self-harm prevention; however, given that their benefits are marginal (Fox et al., 2020), establishing strategies for preventing suicide by addressing other conditions such as chronic pain may be promising. For example, among 250 consecutive patients admitted to a 4-week, group-based chronic pain management program, a significant reduction in SI was observed after treatment, and the authors found that individuals with high SI had greater pain catastrophizing and self-perceived burden (Kowal et al., 2014).

However, to establish an effective suicide prevention strategy through chronic pain management, it is necessary to identify the various pathways and related factors through which chronic pain can contribute to suicidal behavior (SB). These efforts may help identify sub-vulnerable groups with chronic pain in need of suicidality screening and treatment. Therefore, this systematic review aimed to comprehensively investigate the prevalence and associated factors of SBs including SI and suicide attempt (SA) in individuals with chronic pain. Based on the findings described above, the underlying hypothesis of this study was that individuals with chronic pain would be susceptible to SB. Accordingly, the first research question of this review was (1) “*What is the prevalence of SB among individuals with chronic pain?*” Although this study aimed to determine the prevalence of SB among individuals with chronic pain, the results are unlikely to demonstrate a causal relationship between chronic pain and SB. Instead, we sought to summarize the factors associated with SB found through regression analysis in the population. Accordingly, the second research question of this review was (2) “*What are the factors associated with the presence of SB among individuals with chronic pain?*”

## 2. Methods

This systematic review complied with the Preferred Reporting Items for Systematic Reviews and Meta-Analyses (PRISMA) 2020 statement (Page et al., 2021) (Supplementary file 1). In addition, the protocol of this systematic review was registered in Open Science Framework.^1^ This registry is one of the registers where systematic reviews can be prospectively registered (Pieper and Rombey, 2022). The protocol of this systematic review was registered on 11 June 2022. After registration in the registry, there was no amendment to the protocol.

### 2.1. Search strategy

Comprehensive searches were performed by a researcher (CYK) in the following five electronic bibliographic databases: MEDLINE via PubMed, Cochrane Library, EMBASE via Elsevier, Cumulative Index to Nursing and Allied Health Literature via EBSCO, and PsycARTICLES via ProQuest. The search strategy was reviewed by experts in literature search. The search strategies and search results for each database are presented in Supplementary file 2. In addition, the researcher (CYK) reviewed the reference lists of the relevant review articles and manually searched on Google Scholar to identify potentially missing literature. The search date was October 4, 2022, and all relevant studies published up to the search date were reviewed.

### 2.2. Eligibility criteria

(a) Participants/population: Patients with chronic pain, defined as experiencing pain for more than 3 months (Treede et al., 2015). No restrictions were placed on the cause of the pain. Studies were excluded if the duration of pain of the participants was unclear or if the studies included both chronic and acute pain patients but did not analyze them separately. There were no restrictions on the participants’ age, race/ethnicity, or sex/gender. (b) Interventions/exposures: Not applicable. (c) Comparators/controls: Not applicable. (d) Outcomes: In this review, SB includes SI and SA, but not nonsuicidal self-injury. The primary outcome of this review was any validated measure of individual components of SB (i.e., SI and SA) including the Beck Scale for Suicide Ideation (BSSI) (Beck et al., 1979). Other measures of SB were considered as secondary outcomes. Cases in which the prevalence of SB was presented following an interview with the researcher or clinician, without the use of the screening instrument, were allowed. However, studies that did not provide the prevalence of SB or presented only an unclear percentage, which made it difficult to estimate the raw data, were excluded. (e) Study type: Only observational studies including cross-sectional and longitudinal observational studies were allowed. Animal experiments, review articles, and interventional studies were excluded. There were no restrictions on the format of the research, and gray literature such as dissertations and conference abstracts were accepted. In addition, there were no restrictions on the publication year and publication language.

### 2.3. Study selection

The study selection process was conducted in a two-step process. The first step was the screening step, where two researchers (CYK and BL) independently reviewed the titles and abstracts of the initially retrieved documents and selected potentially relevant documents for the second step. Documents whose potential relevance could not be identified from the titles and abstracts at this step were also selected for the second step. In the second step, the full text of potentially relevant documents was further independently reviewed by the two researchers (CYK and BL). For documents excluded in the second step, the reason for the exclusion was individually stated. Through the two-step process, studies were finally selected for inclusion. During this process, when disagreements between the researchers arose, they were resolved through consensus. Software EndNote X20 (Clarivate Analytics, Philadelphia, PA, United States) was used to manage the citations of the searched documents.

### 2.4. Data collection

Data extraction was conducted using a pre-defined and pilot-tested Excel form (Microsoft 365, DC; Microsoft, Redmond, WA, USA). Information extracted from included studies was as follows: country of 1st author; study settings; sample size; sex, race/ethnicity, mean age, clinical conditions, duration of pain of the participants; prevalence of SB; and factors explaining the association between chronic pain and SB through regression analysis. Data extraction was conducted by two independent researchers (CYK and BL), and discrepancies were resolved through consensus. If the data were insufficient or ambiguous, an inquiry was sent through e-mail to the corresponding author of the study.

### 2.5. Assessment of methodological quality

To assess the methodological quality of the included observational studies, the Strengthening the Reporting of Observational Studies in Epidemiology (STROBE) statement was used (Von Elm et al., 2007). Among the 22-item checklist of this tool (Von Elm et al., 2007), six modified criteria were used for the assessment of the methodological quality of the included studies as follows (Allagh et al., 2015; Ha et al., 2021): (a) *Is there any clear description of the study settings?*; (b) *Is sufficient information about the participants presented?*; (c) Are *validated criteria (i.e., assessment tool) for SB used?*; (d) *Is there information on whether the participants provided informed consent?*; (e) *Is there any description of consecutive participants?*; and (f) *Can the results be generalized to individuals with chronic pain?*

### 2.6. Data analysis

All included studies were subjected to qualitative analysis. A meta-analysis was conducted to quantify the prevalence of SB when two or more studies reported the same outcome of SB. SB was divided into its individual components, SI and SA, and analyzed. For the meta-analysis, STATA/MP software version 16 (StataCorp LLC, College Station, TX, United States) with the command “Metaprop” was used (Nyaga et al., 2014). This command allows the quantitative synthesis of binomial data, which may be used for pooled prevalence estimation (Nyaga et al., 2014). Because this review did not limit specific types of chronic pain in the eligibility criteria, potential clinical heterogeneity among the included studies was inevitable. Given the potential heterogeneity of the included studies, a random-effects model was used to perform the meta-analysis in this review. The estimated prevalence of each SB and the 95% confidence interval (CI) were calculated. An *I*^2^ value greater than 50 and 75% was considered to indicate substantial and high heterogeneity, respectively. To account for potential heterogeneity, subgroup analysis was performed for studies that used validated tools for assessing SB and studies that did not. In addition, subgroup analysis according to population type was conducted.

## 3. Results

### 3.1. Study selection

**Figure 1:**
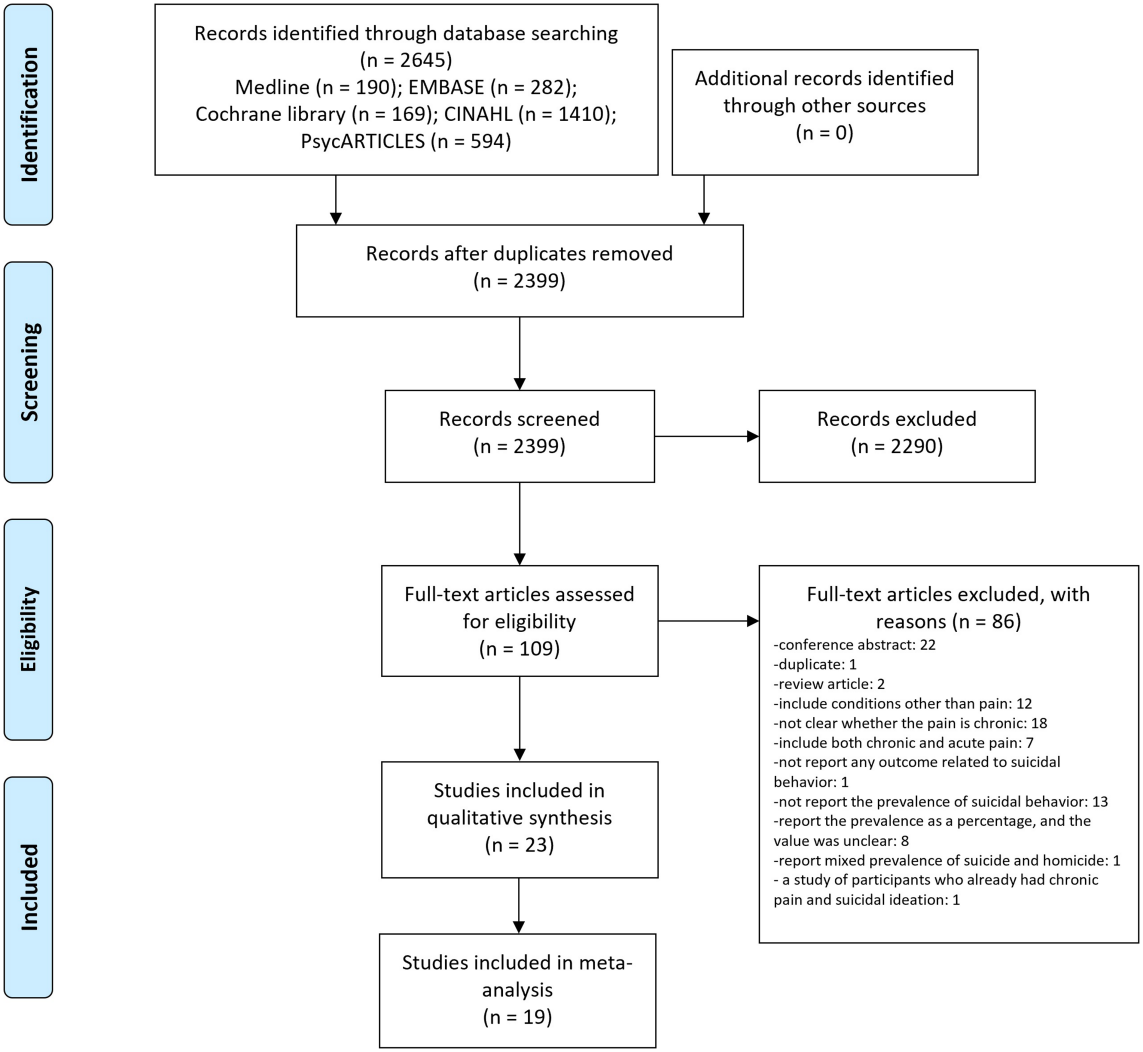
PRISMA flow diagram of this review. CINAHL, cumulative index to nursing and allied health literature.

Of the 2,645 documents initially retrieved, 246 duplicates were removed. The titles and abstracts of 2,399 documents were reviewed in the first step, and 2,290 documents not relevant to this review were excluded. In the second step, the full text of the remaining 109 documents was reviewed, and 86 of them were excluded, which included conference abstracts (*n* = 22), duplicates (*n* = 1), review articles (*n* = 2), studies including conditions other than pain (*n* = 12), studies that were unclear on whether the pain is chronic (*n* = 18), studies that included both chronic and acute pain (*n* = 7), a study that did not report any outcome related to SB (*n* = 1), studies that did not report the prevalence of SB (*n* = 13), studies that reported the prevalence but only presented it as a percentage with an unclear value (*n* = 8), a study reporting a mixed prevalence of suicide and homicide (*n* = 1), and a study of participants who already had chronic pain and SI (*n* = 1) (Supplementary file 3). Therefore, a total of 23 studies (*N* = 3,807,687) (Smith et al., 2004; Ratcliffe et al., 2008; Poole et al., 2009; Kanzler et al., 2012; Dutta et al., 2013; Cheatle et al., 2014; Ciaramella and Poli, 2015; Im et al., 2015; Campbell et al., 2015a,b, 2016; Bertoli and de Leeuw, 2016; Bromberg et al., 2017; Ciaramella, 2017; Wilson et al., 2017; Blakey et al., 2018; Lewcun et al., 2018; Vaegter et al., 2019; Abdelghani et al., 2020; Androulakis et al., 2021; Rojas et al., 2021; Wang et al., 2021; Song et al., 2022) were included in this review. Of these studies, 19 studies (*N* = 3,312,343) (Smith et al., 2004; Ratcliffe et al., 2008; Poole et al., 2009; Kanzler et al., 2012; Dutta et al., 2013; Cheatle et al., 2014; Campbell et al., 2015a,b, 2016; Bertoli and de Leeuw, 2016; Bromberg et al., 2017; Ciaramella, 2017; Wilson et al., 2017; Blakey et al., 2018; Abdelghani et al., 2020; Androulakis et al., 2021; Rojas et al., 2021; Song et al., 2022) were included in the meta-analysis. The study selection process is presented as a PRISMA flow diagram (Figure 1).

### 3.2. Characteristics of the included studies

The included studies were conducted in nine countries in the following order of decreasing frequency: United States (*n* = 11, 47.83%); Australia (*n* = 3, 13.04%); Canada and Italy (*n* = 2, 8.70%, each); United Kingdom, India, Denmark, Egypt, and Taiwan (*n* = 1, 4.35%, each). The average sample size of the included studies was 165,552, with a total of 3,807,687 participants. In 10 studies (Smith et al., 2004; Poole et al., 2009; Kanzler et al., 2012; Cheatle et al., 2014; Bromberg et al., 2017; Blakey et al., 2018; Lewcun et al., 2018; Abdelghani et al., 2020; Androulakis et al., 2021; Song et al., 2022), information on the race or ethnicity of the participants was presented, and all of them included patients of various ethnicities. Most of the studies did not have age restrictions for the participants; however, two studies (Bromberg et al., 2017; Lewcun et al., 2018) included only adolescent participants. In addition, four studies (Im et al., 2015; Blakey et al., 2018; Androulakis et al., 2021; Song et al., 2022) included only veterans. Most of the studies targeted multiple chronic pain conditions regardless of type; however, three studies (Bertoli and de Leeuw, 2016; Lewcun et al., 2018; Wang et al., 2021) were limited to certain types of chronic pain. The most frequently reported outcome of SB was past 2-week SI, which was reported in eight studies (Poole et al., 2009; Kanzler et al., 2012; Dutta et al., 2013; Cheatle et al., 2014; Bertoli and de Leeuw, 2016; Bromberg et al., 2017; Blakey et al., 2018; Lewcun et al., 2018), followed by current SI [*n* = 6 (Smith et al., 2004; Ciaramella and Poli, 2015; Im et al., 2015; Bertoli and de Leeuw, 2016; Rojas et al., 2021; Song et al., 2022)], lifetime SA [*n* = 6 (Smith et al., 2004; Campbell et al., 2015a,b, 2016; Abdelghani et al., 2020; Wang et al., 2021)], lifetime SI [*n* = 5 (Smith et al., 2004; Campbell et al., 2015b, 2016; Abdelghani et al., 2020; Wang et al., 2021)], past year SI [*n* = 4 (Ratcliffe et al., 2008; Campbell et al., 2015a,b, 2016)], and past year SA [*n* = 4 (Ratcliffe et al., 2008; Campbell et al., 2015a,b, 2016)] (Table 1).

**Table 1:** Characteristics of included studies.

Study (Country)	Study setting (Inpatient or Outpatient)/Race or ethnicity	Sample size (M:F)/Mean age (year)	Clinical condition/Pain duration	Outcome on the prevalence of suicidal behavior
Smith et al. (2004) (US)	A tertiary care pain treatment center of the University (outpatient)/White, African-American	153 (56.6% F)/44.7 ± 14.4 (15–93)	Non-malignant pain including LBP, lower extremity pain, abdominal pain, neck pain, upper extremity pain, total body pain/5.2 ± 7.4 year	1. Structured clinical interview for suicide history in chronic pain (42-item structured interview developed by one of the authors): (1) current passive SI; (2) current active SI; (3) current plan to end life; (4) current intent to end life; (5) lifetime passive SI; (6) lifetime active SI; (7) lifetime plan to end life; (8) lifetime intent to end life; (9) lifetime SA
Ratcliffe et al. (2008) (Canada)	Nationally representative sample (NR)/NR	36,984 (16,773:20211)/NR (15-)	Chronic pain including migraine, arthritis or rheumatism, backache, fibromyalgia, others/more than 6 months	1. Questions: (1) past year SI; (2) past year SA
Poole et al. (2009) (UK)	A specialist center (NR)/British, Black Caribbean, Black African, Indian, Pakistani, other Asian	1,227 (467:760)/46.73 ± 11.30	Chronic pain including whole body pain, headache, visceral pain, neuropathic pain/8.8 ± 7.8 year	1. SI item of BDI-II (≥1)
Kanzler et al. (2012) (US)	A clinical health psychology clinic (outpatient)/Caucasian, other	113 (65.2% F)/41.91 ± 13.4 (19–77)	Chronic pain including headaches/migraines, chronic lower back pain, fibromyalgia syndrome, TMD and other myofascial pain, arthritis, CRPS/NR	1. SI item of BDI-II (≥1)
Dutta et al. (2013) (India)	A pain clinic (NR)/NR	476 (195:281)/49.57 (18–90)	Chronic pain including joint pain, back pain, cervical pain, arm or leg pain, generalized body pain, other/49.35 months	1. SI item of PHQ-9
Cheatle et al. (2014) (US)	NR (NR)/Hispanic, White, Black, other	466 (185:278) information on three people was unclear/47.95 ± 12.73	Chronic pain on spine, head, abdomen, extremity, generalized/without SI: 8.55 ± 9.48 yr.; with SI: 10.35 ± 10.11 year	1. Passive or active SI item of BDI-FS
Campbell et al. (2015a) (Australia)	Nationally representative sample (NR)/NR	978 (437:541)/57.5 ± 13.6	Chronic non-cancer pain including arthritis, chronic back/neck problems, frequent headaches/migraines, visceral pain, fibromyalgia/10 year	1. Questions adopted from the WMH-CIDI suicidality module (based on DSM-IV): (1) past year SI; (2) past year SA; (3) lifetime SA; (4) ever self-harm
Campbell et al. (2015b) (Australia)	Nationally representative sample (NR)/NR	3,585 (1,447:2138)/NR (16–85)	Chronic pain including arthritis, migraines, back/neck problems, other/more than 6 months	1. Questions adopted from the WMH-CIDI suicidality module (based on DSM-IV): (1) past year SI; (2) past year suicidal plan; (3) past year SA; (4) lifetime SI; (5) lifetime suicidal plan; (6) lifetime SA
Ciaramella and Poli (2015) (Italy)	A tertiary pain clinic (NR)/NR	LBP: 427 (167:260); other pain: 629 (201:428)/LBP: 60.97 ± 13.75; other pain: 52.66 ± 17.08	Chronic pain including back pain, phantom pain, ischemic pain, postherpetic neuralgia, CRPS, arthrosis, headache, facial pain, fibromyalgia, cancer, and others/LBP: 101.76 ± 106.43 months; other pain: 102.42 ± 132.24 months	1. The structured diagnostic interview for DSM-IV: (1) lifetime suicide risk
Im et al. (2015) (US)	VHA National Patient Care Database and VHA Decision Support System pharmacy and laboratory files (NR)/NR	487,462 (NR)/NR	Veteran with chronic pain who was prescribed opioids/NR	1. Completed or attempted suicide within the first 180 days after opioid prescription
Bertoli and de Leeuw (2016) (US)	An orofacial pain center at the University (NR)/NR	Total: 1241 (145:1096); myofascial pain: 534 (59:475); TMJ pain: 246 (32:214); myofascial + TMJ pain: 461 (54:407)/Total: 35.76 ± 12.6; myofascial pain: 37.32 ± 11.9; TMJ pain: 32.24 ± 13.0; myofascial + TMJ pain: 34.76 ± 12.9	Chronic TMD/Total: 50.50 ± 70.5 months; myofascial pain: 48.50 ± 67.8 months; TMJ pain: 44.92 ± 67.9 months; myofascial + TMJ pain: 55.80 ± 74.6 months	1. SCL-90-R item: (1) “thoughts of ending your life” (item 15); (2) “feeling hopeless about the future” (item 54); (3) “thoughts of death or dying” (item 59)
Campbell et al. (2016) (Australia)	Community pharmacies across Australia (NR)/NR	1,514 (672:842)/58 ± 13.7	Chronic non-cancer pain/10 year	1. Questions adopted from the WMH-CIDI suicidality module (based on DSM-IV): (1) past year SI; (2) past year suicidal plan; (3) past year SA; (4) lifetime SI; (5) lifetime suicidal plan; (6) lifetime SA
Bromberg et al. (2017) (US)	A multidisciplinary pediatric chronic pain clinic (NR)/Caucasian, other	95 (NR)/15.6 ± 2.6	Chronic pain/more than 3 months	1. MFQ (past 2 weeks): (1) five items on SI (≥1)
Ciaramella (2017) (Italy)	GIIM (inpatient)/NR	Total: 515 (177:338); with mood disorders: 381 (118:263); without psychiatric disorder: 134 (59:75)/with mood disorders: 58.51 ± 15.58; without psychiatric disorder: 59.23 ± 16.38	Chronic pain/more than 3 months	1. DSM-IV: (1) current SI
Wilson et al. (2017) (Canada)	A publicly funded hospital (outpatient)/NR	282 (NR)/48.23 ± 11.37	Chronic pain on back, limbs, neck/8.45 ± 8.51 year	1. BSSI (≥1)
Blakey et al. (2018) (US)	PDMH (NR)/White, other	667 (540:127)/37.82 ± 10.52	Chronic pain including headaches, sprains, toothaches/more than 7 months	1. BSSI (≥3) 2. SI item of BDI-II (≥1)
Lewcun et al. (2018) (US)	A tertiary pediatric amplified pain clinic (NR)/White, Black/African American, Asian, American Indian/Alaskan Native, Hispanic, other	453 (83:369)/14.34 ± 1.83	Amplified musculoskeletal pain syndrome/24.53 ± 27.66 months	1. CDI: (1) past 2 weeks passive SI (item 9) (=1); (2) past 2 weeks active SI (item 9) (=2)
Vaegter et al. (2019) (Denmark)	A multidisciplinary pain clinic (NR)/NR	6,142 (2,201:3941)/48.2 ± 14.2	Non-malignant chronic pain/NR	1. ICD code: (1) suicides (X60-X84 and Y870)
Abdelghani et al. (2020) (Egypt)	NIMHCPES (NR)/Asian, Hispanic, Black, White	5301 (NR)/with SI: 42.1 ± 14.8; without SI: 47.2 ± 16.9; with SA: 40.1 ± 14.2; without SA: 43.1 ± 15.0	NR/with SI: 6.8 ± 7.4 year.; without SI: 8.7 ± 12.2 year.; with SA: 7.0 ± 8.0 year.; without SA: 6.5 ± 6.9 year	1. Self-report: (1) lifetime SI; (2) lifetime SA
Androulakis et al. (2021) (US)	Multiple clinical centers and systems across the nation (inpatient and outpatient)/Asian, Black or African, American, White, other	3,247,621 (NR)/NR (18–95)	Chronic pain including headache, neck pain, back pain, other/more than 3 months	1. ICD code: (1) SA (specified intent of self-harm)
Rojas et al. (2021) (US)	A private multispecialty orthopedic clinic (NR)/NR	27 (14:13)/56.3 ± 18.2	NR/more than 3 months	1. AEQ: (1) current SI (“life is hardly worth living with pain like this”) (≥1)
Wang et al. (2021) (Taiwan)	The Headache Clinic of Taipei Veterans Hospital (NR)/NR	Total: 603 (118:485); with medication overuse headache: 283 (55:228); without medication overuse headache: 320 (63:257)/Total: 42.0 ± 12.2; with medication overuse headache: 41.2 ± 12.7; without medication overuse headache: 42.8 ± 11.7	Chronic migraine with or without medication overuse headache/more than 1 months	1. Questions: (1) lifetime SI; (2) lifetime SA
Song et al. (2022) (US)	Post-9/11 Veterans database (inpatient and outpatient)/Black, White, Hispanic, other	10,717 (10,017:700)/(NR)	Chronic pain including back/neck pain, other musculoskeletal pain, and headache, with mild TBI/NR	1. ICD code: (1) SI; (2) SA

AEQ, Avoidance Endurance Questionnaire; BDI-FS, Beck Depression Inventory-Fast Screen; BDI, Beck Depression Inventory; BSSI, Beck Scale for Suicidal Ideation; CDI, Children’s Depression Inventory; CRPS, complex regional pain syndrome; DSM-IV, Diagnostic and Statistical Manual of Mental Disorders, fourth edition; GIIM, the Gift Institute for Integrative Medicine; ICD, International Classification of Diseases; LBP, low back pain; MFQ, Mood and Feeling Questionnaire; NIMHCPES, National Institute of Mental Health Collaborative Psychiatric Epidemiology Surveys; NR, not reported; PDMH, Post-Deployment Mental Health Study; PHQ, Patient Health Questionnaire; SA, suicide attempt; SCL-90-R, Symptom Checklist-90-Revised; SI, suicidal ideation; TBI, traumatic brain injury; TMD, temporomandibular disorders; TMJ, temporomandibular joint; VHA, Veterans Health Administration; WMH-CIDI, World Mental Health Composite International Diagnostic Interview.

### 3.3. Methodological quality assessment

Except for one study (Cheatle et al., 2014), all other included studies (Smith et al., 2004; Ratcliffe et al., 2008; Poole et al., 2009; Kanzler et al., 2012; Dutta et al., 2013; Ciaramella and Poli, 2015; Im et al., 2015; Campbell et al., 2015a,b, 2016; Bertoli and de Leeuw, 2016; Bromberg et al., 2017; Ciaramella, 2017; Wilson et al., 2017; Blakey et al., 2018; Lewcun et al., 2018; Vaegter et al., 2019; Abdelghani et al., 2020; Androulakis et al., 2021; Rojas et al., 2021; Wang et al., 2021; Song et al., 2022) specified their study settings. Most of the included studies (Smith et al., 2004; Ratcliffe et al., 2008; Poole et al., 2009; Kanzler et al., 2012; Dutta et al., 2013; Cheatle et al., 2014; Ciaramella and Poli, 2015; Im et al., 2015; Campbell et al., 2015a,b, 2016; Bertoli and de Leeuw, 2016; Bromberg et al., 2017; Ciaramella, 2017; Blakey et al., 2018; Lewcun et al., 2018; Vaegter et al., 2019; Abdelghani et al., 2020; Androulakis et al., 2021; Rojas et al., 2021; Wang et al., 2021; Song et al., 2022) provided demographic information such as the age and sex/gender of the participants. However, in one study (Wilson et al., 2017), the sex/gender information of the participants was missing; thus, the question regarding sufficient information was answered as “no”. Five studies (Ciaramella and Poli, 2015; Campbell et al., 2015a,b, 2016; Ciaramella, 2017) using structured interviews based on the Diagnostic and Statistical Manual of Mental Disorders-Fourth Edition (DSM-IV) and two studies (Wilson et al., 2017; Blakey et al., 2018) using the BSSI for the assessment of SB were considered to have used a validated SB assessment tool. In one study (Im et al., 2015), the SB assessment tool was not specified. Four studies (Smith et al., 2004; Ratcliffe et al., 2008; Abdelghani et al., 2020; Wang et al., 2021) using unvalidated interviews or questionnaires, eight studies (Poole et al., 2009; Kanzler et al., 2012; Dutta et al., 2013; Cheatle et al., 2014; Bertoli and de Leeuw, 2016; Bromberg et al., 2017; Lewcun et al., 2018; Rojas et al., 2021) using validated tools to assess other psychiatric symptoms (e.g., depression) but not SBs, and three studies (Vaegter et al., 2019; Androulakis et al., 2021; Song et al., 2022) using the International Classification of Diseases (ICD) codes to estimate SB were considered as not using validated assessment tools for SB. Twelve studies comprising retrospective chart reviews or retrospective cohort studies (e.g., nationally representative sample) (Smith et al., 2004; Ratcliffe et al., 2008; Kanzler et al., 2012; Cheatle et al., 2014; Im et al., 2015; Campbell et al., 2015a,b, 2016; Ciaramella, 2017; Abdelghani et al., 2020; Androulakis et al., 2021; Song et al., 2022) were evaluated as ‘not applicable’ for the question regarding informed consent from the participants. Among the other studies, informed consent from the participants was mentioned in nine studies (Dutta et al., 2013; Ciaramella and Poli, 2015; Bertoli and de Leeuw, 2016; Bromberg et al., 2017; Wilson et al., 2017; Blakey et al., 2018; Lewcun et al., 2018; Rojas et al., 2021; Wang et al., 2021), and the remaining two studies (Poole et al., 2009; Vaegter et al., 2019) did not provide information on informed consent. Five studies (Ratcliffe et al., 2008; Im et al., 2015; Campbell et al., 2015a,b; Abdelghani et al., 2020) using nationally representative samples or national patient care databases were evaluated as “not applicable” for the question regarding consecutive participants. Among the other studies, there was a description of consecutive participants in only five studies (Smith et al., 2004; Bertoli and de Leeuw, 2016; Ciaramella, 2017; Wilson et al., 2017; Wang et al., 2021). For the question regarding the generalizability of the study results, the following studies targeting a specific population were evaluated as ‘no’: four studies (Im et al., 2015; Blakey et al., 2018; Androulakis et al., 2021; Song et al., 2022) on veterans, two studies (Bromberg et al., 2017; Lewcun et al., 2018) on adolescents, and three studies (Bertoli and de Leeuw, 2016; Lewcun et al., 2018; Wang et al., 2021) on certain types of chronic pain (Table 2).

**Table 2:** Methodological quality of included studies.

Study	(a) Are there any clear descriptions of the study settings?	(b) Is sufficient information about the participants presented?	(c) Validated criteria (i.e., assessment tool) for suicidal behavior used?	(d) Is there a description of whether the participants provided informed consent?	(e) Are there any descriptions of consecutive participants?	(f) Can these results be generalized to individuals with chronic pain?
Smith et al. (2004)	Y	Y	N	NA	Y	Y
Ratcliffe et al. (2008)	Y	Y	N	NA	NA	Y
Poole et al. (2009)	Y	Y	N	N	N	Y
Kanzler et al. (2012)	Y	Y	N	NA	N	Y
Dutta et al. (2013)	Y	Y	N	Y	N	Y
Cheatle et al. (2014)	N	Y	N	NA	N	Y
Campbell et al. (2015a)	Y	Y	Y	NA	NA	Y
Campbell et al. (2015b)	Y	Y	Y	NA	NA	Y
Ciaramella and Poli (2015)	Y	Y	Y	Y	N	Y
Im et al. (2015)	Y	Y	NR	NA	NA	N
Bertoli and de Leeuw (2016)	Y	Y	N	Y	Y	N
Campbell et al. (2016)	Y	Y	Y	NA	N	Y
Bromberg et al. (2017)	Y	Y	N	Y	N	N
Ciaramella (2017)	Y	Y	Y	NA	Y	Y
Wilson et al. (2017)	Y	N	Y	Y	Y	Y
Blakey et al. (2018)	Y	Y	Y	Y	N	N
Lewcun et al. (2018)	Y	Y	N	Y	N	N
Vaegter et al. (2019)	Y	Y	N	N	N	Y
Abdelghani et al. (2020)	Y	Y	N	NA	NA	Y
Androulakis et al. (2021)	Y	Y	N	NA	N	N
Rojas et al. (2021)	Y	Y	N	Y	N	Y
Wang et al. (2021)	Y	Y	N	Y	Y	N
Song et al. (2022)	Y	Y	N	NA	N	N

N, no; NA, not applicable; NR, not reported, Y, yes.

### 3.4. Prevalence of SB among individuals with chronic pain

#### 3.4.1. Mixed sample

Based on the meta-analysis results, the pooled prevalence of SI in a mixed sample with different types of pain was as follows: currently, 16.70% (95% CI: 10.68–23.72%); in the past 2 weeks, 25.87% (95% CI: 18.09–34.50%); in the past year, 10.46% (95% CI: 4.34–18.81%); and lifetime, 28.90% (95% CI: 17.95–41.26%). In addition, the pooled prevalence of SA in this population was as follows: in the past year, 1.32% (95% CI: 0.62–2.27%) and lifetime, 10.83% (95% CI: 5.72–17.30%). In subgroup analysis according to whether validated assessment tools for SB were used, the pooled prevalence in studies using validated tools was 1.7–3.4 times higher than that in studies using unvalidated tools, except for past 2-week SI. The pooled prevalence of past 2-week SI in studies using unvalidated tools was 1.9 times higher than that in studies using validated tools for SB (28.67% vs. 15.29%). In subgroup analysis according to the population type, the pooled prevalence of current SI in studies without a specific population was 2.2 times higher than that in studies on veterans (20.73% vs. 9.24%). Moreover, for past 2-week SI, the pooled prevalence was the highest in studies on adolescents, followed by studies without a specific population and studies on veterans (34.74% vs. 27.48% vs. 15.29%) (Table 3).

**Table 3:** Meta-analysis of the prevalence of suicidal behavior among individuals with chronic pain.

Pain conditions		No. of dataset	No. of participants	Prevalence	95% CIs	*I*^2^ value
1. Mixed sample						
(1) current SI	Total	6	12,361	16.70%	10.68 to 23.72%	96.09%
Subgroup analysis 1	Validated tool	4	1,617	18.96%	11.73 to 27.43%	93.48%
	Unvalidated tool	2	10,744	8.34%	7.79 to 8.90%	NA
Subgroup analysis 2	Veteran	2	11,384	9.24%	8.72 to 9.78%	NA
	Unspecified	4	977	20.73%	11.84 to 31.27%	90.81%
(2) past 2 weeks SI	Total	6	3,029	25.87%	18.09 to 34.50%	95.80%
Subgroup analysis 1	Validated tool	1	667	15.29%	12.76 to 18.22%	NA
	Unvalidated tool	5	2,362	28.67%	22.61 to 35.1%	88.76%
Subgroup analysis 2	Adolescent	1	95	34.74%	25.93 to 44.74%	NA
	Veteran	1	667	15.29%	12.76 to 18.22%	NA
	Unspecified	4	2,267	27.48%	20.72 to 34.80%	91.50%
(3) past year SI	Total^§ns^	4	43,070	10.46%	4.34 to 18.81%	99.53%
Subgroup analysis 1	Validated tool	3	6,086	13.30%	3.17 to 28.86%	NA
	Unvalidated tool	1	36,984	3.93%	3.74 to 4.14%	NA
(4) lifetime SI	Total^§ns^	4	10,553	28.90%	17.95 to 41.26%	99.32%
Subgroup analysis 1	Validated tool	3	5,252	32.84%	14.70 to 54.13%	NA
	Unvalidated tool	1	5,301	18.56%	17.54 to 19.63%	NA
(5) past year SA	Total^§ns^	4	43,070	1.31%	0.62 to 2.29%	94.71%
Subgroup analysis 1	Validated tool	3	6,086	1.66%	0.54 to 3.38%	NA
	Unvalidated tool	1	36,984	0.62%	0.55 to 0.71%	NA
(6) lifetime SA	Total^§ns^	5	11,537	10.83%	5.72 to 17.30%	98.83%
Subgroup analysis 1	Validated tool	4	6,236	11.87%	4.34 to 22.40%	98.99%
	Unvalidated tool	1	5,301	7.00%	6.34 to 7.72%	NA
2. Headache						
(1) SA	Total^¶unv§vet^	10	2,549,807	0.14%	0.14 to 0.15%	0.00%
*3. Musculoskeletal pain*						
(1) past 2 weeks SI	Total^¶unv^	3	1,233	19.25%	14.24 to 24.82%	NA
Subgroup analysis 2	Adolescent	1	453	19.87%	16.45 to 23.79%	NA
	Not specified	2	780	20.42%	17.65 to 23.33%	NA
(2) past year SI	Total^¶unv§ns^	2	16,642	5.02%	4.69 to 5.36%	NA
(3) SA	Total^¶unv§vet^	10	3,663,434	0.14%	0.13 to 0.15%	69.62%
(4) past year SA	Total^¶unv§ns^	2	8,739	0.99%	0.79 to 1.21%	NA
3–1. Backache						
(1) SA	Total^¶unv§vet^	10	1,839,705	0.12%	0.12 to 0.13%	0.00%

CI, confidence interval; NA, not applicable; SA, suicide attempt; SI, suicidal ideation.

^¶unv^As all included studies used an unvalidated suicidal behavior assessment tool, the subgroup analysis 1 was not possible.

^§ns^As all included studies included population types not specified, the subgroup analysis 2 was not possible.

^§vet^As all included studies included veterans, the subgroup analysis 2 was not possible.

#### 3.4.2. Chronic headache

A meta-analysis of the prevalence of SA in chronic headache was conducted based on a 10-year dataset (2001 to 2010) provided in a study using an unvalidated tool for SB (Androulakis et al., 2021). The meta-analysis found that the pooled prevalence of SA among veterans with chronic headache was 0.14% (95% CI: 0.14–0.15%) (Table 3).

#### 3.4.3. Chronic musculoskeletal pain

Based on the meta-analysis results, the pooled prevalence of SB in patients with chronic musculoskeletal pain was as follows: past 2-week SI, 19.25% (95% CI: 14.24–24.82%); past year SI, 5.02% (95% CI: 4.69–5.36%); SA without a specified duration, 0.14% (95% CI: 0.13–0.15%); and past year SA, 0.99% (95% CI: 0.79–1.21%). Subgroup analysis was available only for past 2-week SI and did not show a difference between studies on adolescents and studies without a specific population (19.87% vs. 20.42%). A meta-analysis of the prevalence of SA in chronic backache (a type of chronic musculoskeletal pain) was conducted based on a 10-year dataset (2001 to 2010) provided in a study using an unvalidated tool for SB (Androulakis et al., 2021). The meta-analysis found that the pooled prevalence of SA among veterans with chronic backache was 0.12% (95% CI: 0.12–0.13%) (Table 3).

### 3.5. Factors explaining the association between chronic pain and SB

**Figure 2:**
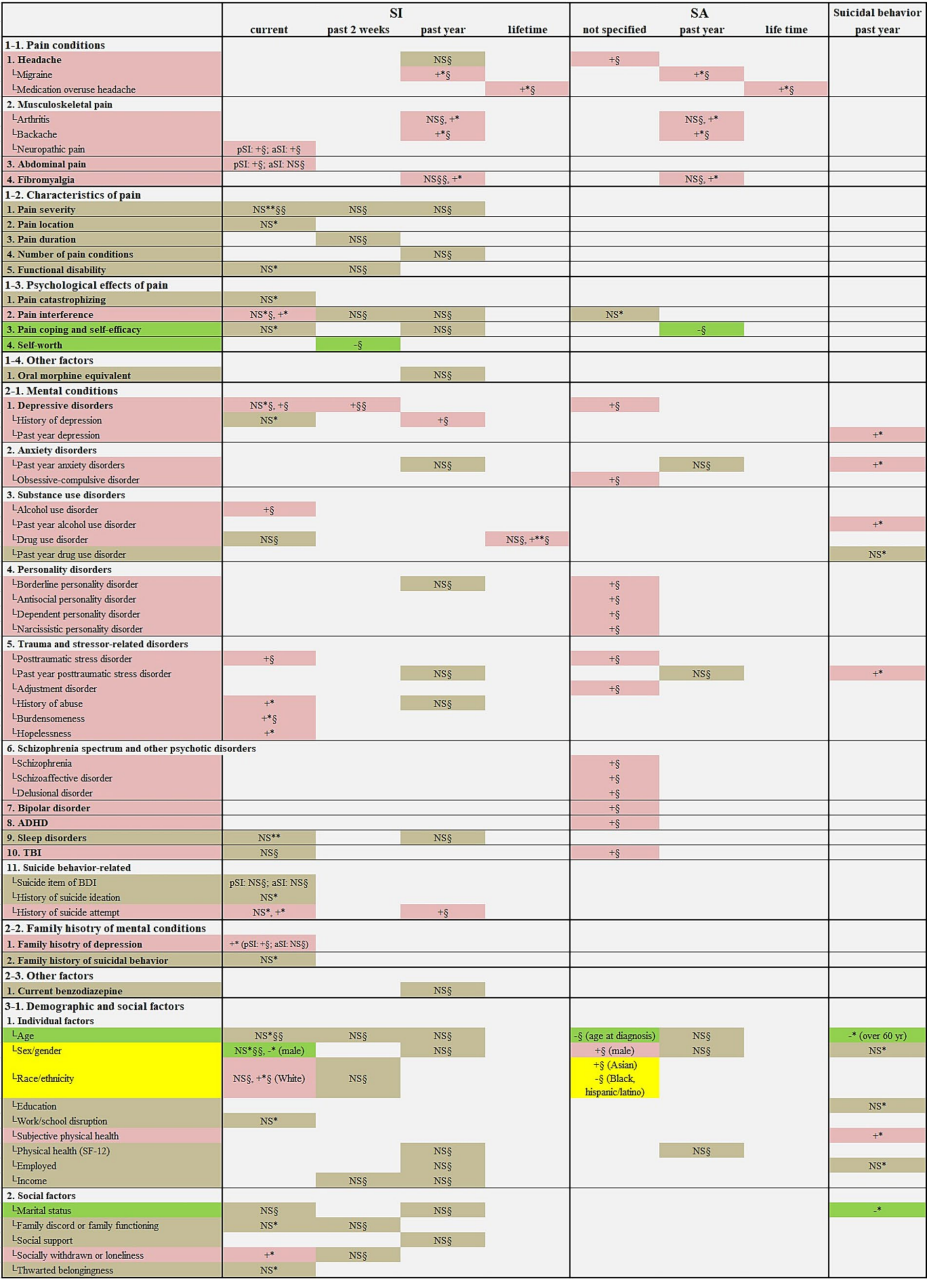
Factors explaining the association between chronic pain and suicidal behavior. ADHD, attention-deficit/hyperactivity disorder; aSI, active suicidal ideation; NS, not significant; pSI, passive suicidal ideation; SA, suicide attempt; SF-12, the 12-Item Short Form Health Survey; SI, suicidal ideation; TBI, traumatic brain injury. Regarding the color of cell shading, red (symbol, “+”) indicates factors significantly associated with an increased risk of suicidal behavior; green (symbol, “−”) indicates factors significantly associated with reduced risk of suicidal behavior; and gray (symbol, “NS”) indicates factors not significantly associated with risk of suicidal behavior. “*” indicates the regression analysis result without adjusting for psychiatric symptoms, and “§” indicates the regression analysis result with psychiatric symptoms such as depression being adjusted. The number of “*” or “§” refers to the number of studies reporting that result. For example, “+§§” means that two regression analyses, adjusting for psychiatric symptoms, reported statistically significant associations with a higher risk of suicidal behavior.

Factors explaining the association between chronic pain and SB, which were identified through regression analysis, were examined. A total of 64 factors were investigated in the included studies, and no statistically significant effects were observed for 21 factors. The remaining 43 factors (67.19%) were found to have statistically significant positive or negative effects on the presence of SB among individuals with chronic pain in at least one study. Of the 43 factors, 37 factors (86.05%) were significantly associated with an increase in SB, 4 factors (9.30%) were significantly associated with a decrease in SB, and the remaining 2 factors (4.65%) were significantly associated with both an increase and a decrease in SB. Clinical factors significantly associated with increased SB in chronic pain patients included pain conditions (e.g., migraine, neuropathic pain, and fibromyalgia) and mental conditions (e.g., depressive disorders, borderline personality disorder, and posttraumatic stress disorder). In addition, some demographic and social factors such as race/ethnicity (White and Asian), poor subjective physical health, and social withdrawal were significantly associated with increased SB in individuals with chronic pain. On the other hand, pain coping and self-efficacy, age (older), race/ethnicity (Black and Hispanic/Latino), and marriage showed potentially protective effects against SB in individuals with chronic pain. Among individuals with chronic pain, sex (male) was associated with a lower prevalence of current SI but a higher prevalence of SA (Figure 2, Supplementary file 4).

## 4. Discussion

### 4.1. Principal findings

This systematic review was conducted to investigate the prevalence of SB and its associated factors in individuals with chronic pain. After a comprehensive search, 23 relevant observational studies (Smith et al., 2004; Ratcliffe et al., 2008; Poole et al., 2009; Kanzler et al., 2012; Dutta et al., 2013; Cheatle et al., 2014; Ciaramella and Poli, 2015; Im et al., 2015; Campbell et al., 2015a,b, 2016; Bertoli and de Leeuw, 2016; Bromberg et al., 2017; Ciaramella, 2017; Wilson et al., 2017; Blakey et al., 2018; Lewcun et al., 2018; Vaegter et al., 2019; Abdelghani et al., 2020; Androulakis et al., 2021; Rojas et al., 2021; Wang et al., 2021; Song et al., 2022) were included in this review. Among them, 19 studies (Smith et al., 2004; Ratcliffe et al., 2008; Poole et al., 2009; Kanzler et al., 2012; Dutta et al., 2013; Cheatle et al., 2014; Campbell et al., 2015a,b, 2016; Bertoli and de Leeuw, 2016; Bromberg et al., 2017; Ciaramella, 2017; Wilson et al., 2017; Blakey et al., 2018; Abdelghani et al., 2020; Androulakis et al., 2021; Rojas et al., 2021; Song et al., 2022) were included in the meta-analysis. Most of the included studies were conducted in Western countries, and only two studies (Dutta et al., 2013; Wang et al., 2021) were conducted in Asian countries. A major factor impairing the methodological quality of the included studies was the lack of use of validated tools for SB. Of the included studies, only seven studies (Ciaramella and Poli, 2015; Campbell et al., 2015a,b, 2016; Ciaramella, 2017; Wilson et al., 2017; Blakey et al., 2018) used validated questionnaires or structured interviews to assess SB. In addition, of the included studies, eight studies (Im et al., 2015; Bertoli and de Leeuw, 2016; Bromberg et al., 2017; Blakey et al., 2018; Lewcun et al., 2018; Androulakis et al., 2021; Wang et al., 2021; Song et al., 2022) targeted specific populations or specific types of chronic pain and thus were at a disadvantage in terms of generalizability. A meta-analysis was conducted on the prevalence of SI and SA among individuals with chronic pain. The results showed that the pooled lifetime prevalence of SI and SA was 28.90 and 10.83%, respectively, in the mixed sample comprising various chronic pain conditions. In addition, the prevalence of SI and SA in this group in the past year was 10.46 and 1.31%, respectively, which was not unusual. The prevalence of SI in the mixed sample ranged from 10 to 25% within a year. Overall, the meta-analysis results showed high statistical heterogeneity (*I*^2^ value of 90% or higher); however, this heterogeneity could not be satisfactorily explained by subgroup analysis. Nevertheless, there was a clear difference in the prevalence of SB in subgroup analyses according to the SB assessment tool or the characteristics of the population. Excluding the pooled prevalence of past 2-week SI, the pooled prevalence rates of current SI, past year SI, lifetime SI, past year SA, and lifetime SA were 1.7 to 3.4 times greater when a validated SB assessment tool was used. In addition, in subgroup analysis according to the characteristics of the subjects in the mixed sample, the overall prevalence of SB was the highest in studies on adolescents, followed by studies without a specific population and studies on veterans. Meta-analysis of the prevalence of SB in individuals with certain pain conditions (headache, musculoskeletal pain, and backache) was available. The pooled prevalence of SA for these individuals was less than 1%, and the prevalence of SI was less than 20%, which was not higher than that for the mixed sample. In the included studies, 43 factors were reported to explain the association between chronic pain and SB, which were identified through regression analysis. Of these factors, most (37/43, 86.05%) were factors associated with an increased risk of SB in individuals with chronic pain; however, some factors were associated with a reduced risk of SB. These potentially protective factors included pain coping and self-efficacy, older age, certain race/ethnicity groups, and marriage.

### 4.2. Clinical implications

A major finding of this systematic review and meta-analysis was the high prevalence of SB among individuals with chronic pain. Specifically, in the mixed sample comprising various chronic pain types, the pooled prevalence of past 2-week SI and lifetime SI reached 25.87 and 28.90%, respectively. Importantly, considering that the pooled prevalence of past 2-week SI was 24.39% among patients with major depressive disorder (MDD), known as a high risk group for suicide, and 1.28% in the non-MDD group (Cai et al., 2021), the pooled prevalence of past 2-week SI among individuals with chronic pain in this review may be considered high. The results of subgroup analyses in this review demonstrated vulnerability to SB in adolescents with chronic pain. A systematic review examining the association between pain and suicide vulnerability in adolescence analyzed 25 observational studies and found that pain could approximately double the risk of suicide of adolescents (Hinze et al., 2019). Importantly, this review shed light on the complex associations between pain and suicidality in adolescents, and most associations became less robust or insignificant after controlling for psychiatric symptoms (Hinze et al., 2019). These findings suggest that psychiatric symptoms including depression may play an important mediating role in the association between pain and suicidality. Regression analyses adjusted for psychiatric symptoms in the studies included in this review identified specific risk factors that may be used as screening tools to determine suicide risk in clinical settings as follows: pain conditions (migraine, medication overuse headache, backache, neuropathic pain, and abdominal pain), mental conditions (depressive disorders, history of depression, obsessive-compulsive disorder, alcohol use disorder, drug use disorder, borderline personality disorder, antisocial personality disorder, dependent personality disorder, narcissistic personality disorder, posttraumatic stress disorder, adjustment disorder, burdensomeness, schizophrenia, schizoaffective disorder, delusional disorder, bipolar disorder, attention-deficit/hyperactivity disorder, traumatic brain injury, history of SA, and family history of depression), sex (male), and race (White) (Figure 2, Supplementary file 4).

Given the high prevalence of SB in patients with chronic pain and the direct and indirect pathways by which chronic pain contributes to suicide (Hooley et al., 2014; Hooten, 2016; Racine, 2018), screening for the suicide risk of individuals with chronic pain may be a promising strategy for reducing the national suicide risk. Overall, subgroup analyses in this review found that the pooled prevalence of SB was higher when validated SB assessment tools were used instead of unvalidated tools. Therefore, compared with unvalidated tools, validated SB assessment tools may be associated with higher sensitivity to SB. Considering that patients who have attempted suicide are more likely to seek treatment in primary care rather than mental health services (Stene-Larsen and Reneflot, 2019) and that somatic symptoms may be a sign of suicide risk (Jeon et al., 2016), appropriate screening of suicide risk using a validated assessment tool with high sensitivity for patients with chronic pain is of public health relevance.

Considering the association between chronic pain and suicide (Hooley et al., 2014; Hooten, 2016; Racine, 2018), the management of chronic pain may be considered for the purpose of reducing the risk of SB in individuals with SB. However, as the use of opioids may contribute to SB in individuals with chronic pain in the context of suicide risk, non-pharmacological therapies may be advantageous for the treatment of chronic pain in these patients (Nestadt and Bohnert, 2020). Promising non-pharmacological therapies include cognitive behavioral therapy, which is commonly used for chronic pain and suicide prevention (Stanley et al., 2009). Moreover, acupuncture, which can reduce suicide risk factors such as chronic pain (Vickers et al., 2018), depression (Armour et al., 2019), and chronic pain-related depression (You et al., 2021), may be considered as one of the promising non-pharmacological therapies for individuals with chronic pain and suicide risk. A recent systematic review examined the effectiveness and safety of acupuncture for SB (Kwon and Lee, 2023). Although definitive conclusions could not be drawn due to the lack of eligible studies, studies of acupuncture involving subjects at risk of suicide have mainly used ear acupuncture (Kwon and Lee, 2023). The potential shared therapeutic mechanisms of ear acupuncture for chronic pain and suicide prevention include stimulation of the auricular branches of the vagus nerve, anti-inflammatory activity, and antioxidant activity (Hou et al., 2015).

In this review, factors associated with the low prevalence of SB in individuals with chronic pain could be considered for a suicide prevention strategy. Potential modifiable protective factors for SB included pain coping and self-efficacy as well as marriage. In particular, self-efficacy plays a role in managing SI and impulses, potentially contributing to the prevention of SB (Czyz et al., 2014). Accordingly, some researchers have suggested that suicide prevention interventions can be provided to adolescents at risk of suicide by enhancing motivation and self-efficacy (Micol et al., 2022). On the other hand, unmodifiable factors included older age and certain race/ethnicity groups, which could be used to identify vulnerability to SB in individuals with chronic pain.

Demoralization and its subcomponent loss of MiL, although not found in this study, are also worth discussing in relation to chronic pain and SB. Importantly, demoralization is relevant to chronic pain as it developed in a somatic context associated to psychic suffering (Clarke and Kissane, 2002). A recent case–control study found that demoralization in patients with chronic pain had a positive correlation with SI, almost as strong as depression (Chytas et al., 2023). Demoralization is often underestimated, but because it is likely an independent risk factor for SB (Costanza et al., 2022), it can be considered an important target to be considered along with chronic pain in the detection of suicide risk. Demoralization may be an important target for screening for suicide risk because it can be identified even in individuals for whom the clinical diagnosis of depression is difficult to define, and is useful because it suggests therapeutic indications, such as meaning-centered psychotherapeutic (Costanza et al., 2022) and interpersonal approaches (Costanza et al., 2020).

### 4.3. Strengths and limitations

This study, for the first time, pooled the prevalence of SB in individuals with chronic pain and presented results highlighting the importance of suicide risk screening in this potentially vulnerable population. Given the high prevalence of chronic pain (Domenichiello and Ramsden, 2019; Murray et al., 2022) and the high suicide rates are still a public health threat (Petrosky et al., 2018; Naghavi, 2019), the findings may contribute to the establishment of strategies for reducing suicide rates in the future. There were some limitations in this systematic review. First, most of the included studies (Smith et al., 2004; Ratcliffe et al., 2008; Poole et al., 2009; Kanzler et al., 2012; Dutta et al., 2013; Cheatle et al., 2014; Ciaramella and Poli, 2015; Im et al., 2015; Campbell et al., 2015a,b, 2016; Bertoli and de Leeuw, 2016; Bromberg et al., 2017; Ciaramella, 2017; Wilson et al., 2017; Blakey et al., 2018; Lewcun et al., 2018; Vaegter et al., 2019; Abdelghani et al., 2020; Androulakis et al., 2021; Rojas et al., 2021) were conducted in Western countries. Among the included studies, 10 studies (Smith et al., 2004; Poole et al., 2009; Kanzler et al., 2012; Cheatle et al., 2014; Bromberg et al., 2017; Blakey et al., 2018; Lewcun et al., 2018; Abdelghani et al., 2020; Androulakis et al., 2021; Song et al., 2022) described the inclusion of patients of various ethnicities; however, relatively few studies on the prevalence of SB in patients with chronic pain in Asian countries were found. Given that a study (Androulakis et al., 2021) included in this review found that Asians were associated with an increased risk of SB in cases of chronic pain, this topic deserves further investigation in Asian countries. This will be a relevant research topic in some Asian countries with high suicide rates, such as South Korea (Ilic and Ilic, 2022). Second, the meta-analysis in this review showed high statistical heterogeneity that could not be explained by subgroup analyses in most cases. This may be attributed to differences in the SB assessment tools, pain conditions, and race/ethnicity and sex of the population. In order to reduce this heterogeneity, certain factors found to explain the relationship between chronic pain and SB in this review can be referenced in the design of future observational studies investigating SB in individuals with chronic pain. Third, among the included studies, no study compared the prevalence of SB between the general population and individuals with chronic pain. Therefore, based on the included studies, we cannot directly conclude that the prevalence of SB in individuals with chronic pain is higher than that in the general population. Fourth, among the included studies, no study longitudinally followed changes in SB in individuals with chronic pain. Therefore, this review did not estimate the direction and trend of longitudinal changes in SBs such as SI or SA in individuals with chronic pain. Also, for the same reason, a causal assessment between the SB-related factors found in this study and the presence of SB was not possible. Therefore, the potentially protective or risk factors should be regarded as potential, and their causality in the context of SB among individuals with chronic pain should be verified in future longitudinal studies. Finally, although recent studies have shown that coronavirus disease 2019 (COVID-19) as a stressful event may affect SB in the general population (Yan et al., 2023), this review was unable to estimate the difference in the prevalence of SB before and after COVID-19 in patients with chronic pain due to a lack of relevant studies. Considering that distress caused by COVID-19 itself may contribute to SB and that limited healthcare visits due to COVID-19 may exacerbate chronic pain and potentially comorbid mental health conditions (John et al., 2022; Tanaka, 2022), this topic should be addressed in the future.

## 5. Conclusion

This systematic review and meta-analysis demonstrated the high prevalence of SB in individuals with chronic pain. Specifically, around 1 in 4 individuals with chronic pain had SI within the last 2 weeks. However, there was considerable statistical heterogeneity in the pooled prevalence of SB in this population. Factors associated with the lower prevalence of SB in this population included pain coping and self-efficacy, age (older), race/ethnicity (Black and Hispanic/Latino), and marriage. The findings of this review may be considered as a public health contribution to address high suicide rates worldwide in the future.

## Data availability statement

The original contributions presented in the study are included in the article/Supplementary material, further inquiries can be directed to the corresponding author.

## Author contributions

C-YK: conceptualization, formal analysis, writing (original draft preparation), and funding acquisition. C-YK and BL: methodology, literature screening/selection, and writing (review and editing). All authors contributed to the article and approved the submitted version.

## Footnotes

^1^https://osf.io/q2n7e

## References

[ref1] Abdelghani M. Ibrahim S. Said A. Foad E. (2020). Can prescription drug use disorder predict suicidality in US adults with chronic pain? A pilot study based on collaborative psychiatric epidemiological surveys. J. Addict. Med. 14, e330–e336. doi: 10.1097/ADM.0000000000000670, PMID: 32487945

[ref2] Allagh K. P. Shamanna B. R. Murthy G. V. Ness A. R. Doyle P. Neogi S. B. . (2015). Birth prevalence of neural tube defects and orofacial clefts in India: a systematic review and meta-analysis. PLoS One 10:e0118961. doi: 10.1371/journal.pone.0118961, PMID: 25768737 PMC4358993

[ref3] Androulakis X. M. Guo S. Zhang J. Sico J. Warren P. Giakas A. . (2021). Suicide attempts in US veterans with chronic headache disorders: a 10-year retrospective cohort study. J. Pain Res. 14, 2629–2639. doi: 10.2147/JPR.S322432, PMID: 34466030 PMC8403028

[ref4] Armour M. Smith C. A. Wang L. Q. Naidoo D. Yang G. Y. Macpherson H. . (2019). Acupuncture for depression: a systematic review and Meta-analysis. J. Clin. Med. 8:1140. doi: 10.3390/jcm8081140, PMID: 31370200 PMC6722678

[ref5] Beck A. T. Kovacs M. Weissman A. (1979). Assessment of suicidal intention: the scale for suicide ideation. J. Consult. Clin. Psychol. 47, 343–352. doi: 10.1037/0022-006X.47.2.343 469082

[ref6] Bertoli E. De Leeuw R. (2016). Prevalence of suicidal ideation, depression, and anxiety in chronic temporomandibular disorder patients. J. Oral Facial Pain Headache 30, 296–301. doi: 10.11607/ofph.1675, PMID: 27792796

[ref7] Blakey S. M. Wagner H. R. Naylor J. Brancu M. Lane I. Sallee M. . (2018). Chronic pain, TBI, and PTSD in military veterans: a link to suicidal ideation and violent impulses? J. Pain 19, 797–806. doi: 10.1016/j.jpain.2018.02.012, PMID: 29526669 PMC6026045

[ref8] Breivik H. Eisenberg E. O'brien T. (2013). The individual and societal burden of chronic pain in Europe: the case for strategic prioritisation and action to improve knowledge and availability of appropriate care. BMC Public Health 13:1229. doi: 10.1186/1471-2458-13-1229, PMID: 24365383 PMC3878786

[ref9] Bromberg M. H. Law E. F. Palermo T. M. (2017). Suicidal ideation in adolescents with and without chronic pain. Clin. J. Pain 33, 21–27. doi: 10.1097/AJP.0000000000000366 26905572

[ref10] Cai H. Xie X. M. Zhang Q. Cui X. Lin J. X. Sim K. . (2021). Prevalence of suicidality in major depressive disorder: a systematic review and Meta-analysis of comparative studies. Front. Psych. 12:690130. doi: 10.3389/fpsyt.2021.690130, PMID: 34603096 PMC8481605

[ref11] Campbell G. Bruno R. Darke S. Degenhardt L. (2015a). Associations of borderline personality with pain, problems with medications and suicidality in a community sample of chronic non-cancer pain patients prescribed opioids for pain. Gen. Hosp. Psychiatry 37, 434–440. doi: 10.1016/j.genhosppsych.2015.05.004, PMID: 26112358

[ref12] Campbell G. Bruno R. Darke S. Shand F. Hall W. Farrell M. . (2016). Prevalence and correlates of suicidal thoughts and suicide attempts in people prescribed pharmaceutical opioids for chronic pain. Clin. J. Pain 32, 292–301. doi: 10.1097/AJP.0000000000000283, PMID: 26295378

[ref13] Campbell G. Darke S. Bruno R. Degenhardt L. (2015b). The prevalence and correlates of chronic pain and suicidality in a nationally representative sample. Aust. N. Z. J. Psychiatry 49, 803–811. doi: 10.1177/0004867415569795, PMID: 25698809

[ref14] Cheatle M. D. Wasser T. Foster C. Olugbodi A. Bryan J. (2014). Prevalence of suicidal ideation in patients with chronic non-cancer pain referred to a behaviorally based pain program. Pain Phys. 17, E359–E367. doi: 10.36076/ppj.2014/17/E359, PMID: 24850117

[ref15] Chytas V. Costanza A. Mazzola V. Luthy C. Bondolfi G. Cedraschi C. (2023). Demoralization and suicidal ideation in chronic pain patients. Psychol. Res. Behav. Manag. 16, 611–617. doi: 10.2147/PRBM.S367461, PMID: 36911045 PMC9997090

[ref16] Ciaramella A. (2017). Mood Spectrum disorders and perception of pain. Psychiatry Q. 88, 687–700. doi: 10.1007/s11126-017-9489-8, PMID: 28091795

[ref17] Ciaramella A. Poli P. (2015). Chronic low Back pain: perception and coping with pain in the presence of psychiatric comorbidity. J. Nerv. Ment. Dis. 203, 632–640. doi: 10.1097/NMD.0000000000000340 26153889

[ref18] Clarke D. M. Kissane D. W. (2002). Demoralization: its phenomenology and importance. Aust. N. Z. J. Psychiatry 36, 733–742. doi: 10.1046/j.1440-1614.2002.01086.x 12406115

[ref19] Costanza A. Amerio A. Aguglia A. Serafini G. Amore M. (2020). Meaning in life and demoralization constructs in light of the interpersonal theory of suicide: a trans-theoretical hypothesis for a cross-sectional study. Psychol. Res. Behav. Manag. 13, 855–858. doi: 10.2147/PRBM.S279829, PMID: 33154680 PMC7605968

[ref20] Costanza A. Chytas V. Piguet V. Luthy C. Mazzola V. Bondolfi G. . (2021). Meaning in life among patients with chronic pain and suicidal ideation: mixed methods study. JMIR Form. Res. 5:e29365. doi: 10.2196/29365, PMID: 34003136 PMC8214181

[ref21] Costanza A. Vasileios C. Ambrosetti J. Shah S. Amerio A. Aguglia A. . (2022). Demoralization in suicide: a systematic review. J. Psychosom. Res. 157:110788. doi: 10.1016/j.jpsychores.2022.110788 35334350

[ref22] Czyz E. K. Bohnert A. S. King C. A. Price A. M. Kleinberg F. Ilgen M. A. (2014). Self-efficacy to avoid suicidal action: factor structure and convergent validity among adults in substance use disorder treatment. Suicide Life Threat. Behav. 44, 698–709. doi: 10.1111/sltb.12101, PMID: 24816132 PMC4229478

[ref23] Dieleman J. L. Baral R. Birger M. Bui A. L. Bulchis A. Chapin A. . (2016). US spending on personal health care and public health, 1996-2013. JAMA 316, 2627–2646. doi: 10.1001/jama.2016.16885, PMID: 28027366 PMC5551483

[ref24] Domenichiello A. F. Ramsden C. E. (2019). The silent epidemic of chronic pain in older adults. Prog. Neuro-Psychopharmacol. Biol. Psychiatry 93, 284–290. doi: 10.1016/j.pnpbp.2019.04.006, PMID: 31004724 PMC6538291

[ref25] Dutta D. Bharati S. Roy C. Das G. (2013). Measurement of prevalence of 'major depressive syndrome' among Indian patients attending pain clinic with chronic pain using PHQ-9 scale. J. Anaesthesiol. Clin. Pharmacol. 29, 76–82. doi: 10.4103/0970-9185.105808, PMID: 23493638 PMC3590548

[ref26] Edwards R. R. Smith M. T. Kudel I. Haythornthwaite J. (2006). Pain-related catastrophizing as a risk factor for suicidal ideation in chronic pain. Pain 126, 272–279. doi: 10.1016/j.pain.2006.07.004, PMID: 16926068

[ref27] Elman I. Borsook D. Volkow N. D. (2013). Pain and suicidality: insights from reward and addiction neuroscience. Prog. Neurobiol. 109, 1–27. doi: 10.1016/j.pneurobio.2013.06.003, PMID: 23827972 PMC4827340

[ref28] Fox K. R. Huang X. Guzmán E. M. Funsch K. M. Cha C. B. Ribeiro J. D. . (2020). Interventions for suicide and self-injury: a meta-analysis of randomized controlled trials across nearly 50 years of research. Psychol. Bull. 146, 1117–1145. doi: 10.1037/bul0000305, PMID: 33119344

[ref29] Ha D.-J. Park J.-H. Jung S.-E. Lee B. Kim M.-S. Sim K.-L. . (2021). The experience of emotional labor and its related factors among nurses in general hospital settings in Republic of Korea: a systematic review and meta-analysis. Sustainability 13:11634. doi: 10.3390/su132111634

[ref30] Hinze V. Crane C. Ford T. Buivydaite R. Qiu L. Gjelsvik B. (2019). The relationship between pain and suicidal vulnerability in adolescence: a systematic review. Lancet Child Adolesc. Health 3, 899–916. doi: 10.1016/S2352-4642(19)30267-6, PMID: 31606322 PMC6842327

[ref31] Hooley J. M. Franklin J. C. Nock M. K. (2014). Chronic pain and suicide: understanding the association. Curr. Pain Headache Rep. 18:435. doi: 10.1007/s11916-014-0435-2 24916035

[ref32] Hooten W. M. (2016). Chronic pain and mental health disorders: shared neural mechanisms, epidemiology, and treatment. Mayo Clin. Proc. 91, 955–970. doi: 10.1016/j.mayocp.2016.04.029 27344405

[ref33] Hou P. W. Hsu H. C. Lin Y. W. Tang N. Y. Cheng C. Y. Hsieh C. L. (2015). The history, mechanism, and clinical application of auricular therapy in traditional Chinese medicine. Evid. Based Complement. Alternat. Med. 2015:495684, 1–13. doi: 10.1155/2015/495684 26823672 PMC4707384

[ref34] Ilic M. Ilic I. (2022). Worldwide suicide mortality trends (2000-2019): a joinpoint regression analysis. World J. Psych. 12, 1044–1060. doi: 10.5498/wjp.v12.i8.1044, PMID: 36158305 PMC9476842

[ref35] Im J. J. Shachter R. D. Oliva E. M. Henderson P. T. Paik M. C. Trafton J. A. (2015). Association of Care Practices with suicide attempts in US veterans prescribed opioid medications for chronic pain management. J. Gen. Intern. Med. 30, 979–991. doi: 10.1007/s11606-015-3220-y, PMID: 25693651 PMC4471010

[ref36] Jeon H. J. Woo J. M. Kim H. J. Fava M. Mischoulon D. Cho S. J. . (2016). Gender differences in somatic symptoms and current suicidal risk in outpatients with major depressive disorder. Psychiatry Investig. 13, 609–615. doi: 10.4306/pi.2016.13.6.609, PMID: 27909451 PMC5128348

[ref37] John H. Lim Y. H. Hong S. J. Jeong J. H. Choi H. R. Park S. K. . (2022). Impact of coronavirus disease 2019 on patients with chronic pain: multicenter study in Korea. Korean J Pain 35, 209–223. doi: 10.3344/kjp.2022.35.2.209, PMID: 35354684 PMC8977200

[ref38] Kanzler K. E. Bryan C. J. Mcgeary D. D. Morrow C. E. (2012). Suicidal ideation and perceived burdensomeness in patients with chronic pain. Pain Pract. 12, 602–609. doi: 10.1111/j.1533-2500.2012.00542.x, PMID: 22429694

[ref39] Kowal J. Wilson K. G. Henderson P. R. Mcwilliams L. A. (2014). Change in suicidal ideation after interdisciplinary treatment of chronic pain. Clin. J. Pain 30, 463–471. doi: 10.1097/AJP.0000000000000003, PMID: 24281291 PMC4014432

[ref40] Kwon C. Y. Lee B. (2023). The effectiveness and safety of acupuncture on suicidal behavior: a systematic review. Healthcare (Basel) 11:955. doi: 10.3390/healthcare11070955 37046882 PMC10094566

[ref41] Lewcun B. Kennedy T. M. Tress J. Miller K. S. Sherker J. Sherry D. D. (2018). Predicting suicidal ideation in adolescents with chronic amplified pain: the roles of depression and pain duration. Psychol. Serv. 15, 309–315. doi: 10.1037/ser0000210, PMID: 30080089

[ref42] Micol V. J. Prouty D. Czyz E. K. (2022). Enhancing motivation and self-efficacy for safety plan use: incorporating motivational interviewing strategies in a brief safety planning intervention for adolescents at risk for suicide. Psychotherapy (Chic.) 59, 174–180. doi: 10.1037/pst0000374, PMID: 34323576 PMC8799764

[ref43] Murray C. B. De La Vega R. Murphy L. K. Kashikar-Zuck S. Palermo T. M. (2022). The prevalence of chronic pain in young adults: a systematic review and meta-analysis. Pain 163, e972–e984. doi: 10.1097/j.pain.0000000000002541, PMID: 34817439

[ref44] Naghavi M. (2019). Global, regional, and national burden of suicide mortality 1990 to 2016: systematic analysis for the global burden of disease study 2016. BMJ 364:l94. doi: 10.1136/bmj.l94 31339847 PMC6598639

[ref45] Nestadt P. S. Bohnert A. S. B. (2020). Clinical perspective on opioids in the context of suicide risk. Focus (Am. Psychiatr. Publ.) 18, 100–105. doi: 10.1176/appi.focus.20200003, PMID: 33162847 PMC7587892

[ref46] Nyaga V. N. Arbyn M. Aerts M. (2014). Metaprop: a Stata command to perform meta-analysis of binomial data. Arch. Public Health 72:39. doi: 10.1186/2049-3258-72-39, PMID: 25810908 PMC4373114

[ref47] Page M. J. Mckenzie J. E. Bossuyt P. M. Boutron I. Hoffmann T. C. Mulrow C. D. . (2021). The PRISMA 2020 statement: an updated guideline for reporting systematic reviews. BMJ 372:n71 33782057 10.1136/bmj.n71 PMC8005924

[ref48] Petrosky E. Harpaz R. Fowler K. A. Bohm M. K. Helmick C. G. Yuan K. . (2018). Chronic pain among suicide decedents, 2003 to 2014: findings from the National Violent Death Reporting System. Ann. Intern. Med. 169, 448–455. doi: 10.7326/M18-0830, PMID: 30208405 PMC6913029

[ref49] Pieper D. Rombey T. (2022). Where to prospectively register a systematic review. Syst. Rev. 11:8. doi: 10.1186/s13643-021-01877-1, PMID: 34998432 PMC8742923

[ref50] Poole H. Bramwell R. Murphy P. (2009). The utility of the Beck depression inventory fast screen (BDI-FS) in a pain clinic population. Eur. J. Pain 13, 865–869. doi: 10.1016/j.ejpain.2008.09.017 19010075

[ref51] Racine M. (2018). Chronic pain and suicide risk: a comprehensive review. Prog. Neuro-Psychopharmacol. Biol. Psychiatry 87, 269–280. doi: 10.1016/j.pnpbp.2017.08.020, PMID: 28847525

[ref52] Ratcliffe G. E. Enns M. W. Belik S. L. Sareen J. (2008). Chronic pain conditions and suicidal ideation and suicide attempts: an epidemiologic perspective. Clin. J. Pain 24, 204–210. doi: 10.1097/AJP.0b013e31815ca2a3, PMID: 18287825

[ref53] Rojas A. M. Worts P. R. Chandler Iii G. S. (2021). Feasibility and clinical utility of assessing behavioral and psychological risk factors in pain management. Pain Physician 24, E1299–e1306. PMID: 34793657

[ref54] Smith M. T. Edwards R. R. Robinson R. C. Dworkin R. H. (2004). Suicidal ideation, plans, and attempts in chronic pain patients: factors associated with increased risk. Pain 111, 201–208. doi: 10.1016/j.pain.2004.06.016, PMID: 15327824

[ref55] Song K. Brintz B. J. Wang C. P. Mcgeary D. D. Mcgeary C. A. Potter J. S. . (2022). Complex pain phenotypes: suicidal ideation and attempt through latent multimorbidity. PLoS One 17:e0267844. doi: 10.1371/journal.pone.0267844, PMID: 35486582 PMC9053801

[ref56] Stanley B. Brown G. Brent D. A. Wells K. Poling K. Curry J. . (2009). Cognitive-behavioral therapy for suicide prevention (CBT-SP): treatment model, feasibility, and acceptability. J. Am. Acad. Child Adolesc. Psychiatry 48, 1005–1013. doi: 10.1097/CHI.0b013e3181b5dbfe, PMID: 19730273 PMC2888910

[ref57] Stene-Larsen K. Reneflot A. (2019). Contact with primary and mental health care prior to suicide: a systematic review of the literature from 2000 to 2017. Scand. J. Public Health 47, 9–17. doi: 10.1177/1403494817746274, PMID: 29207932

[ref58] Tanaka S. (2022). What changes occurred in patients with chronic pain in the early phase of the COVID-19 pandemic? J. Anesth. 36, 332–334. doi: 10.1007/s00540-022-03042-x, PMID: 35107662 PMC8809068

[ref59] Treede R. D. Rief W. Barke A. Aziz Q. Bennett M. I. Benoliel R. . (2015). A classification of chronic pain for ICD-11. Pain 156, 1003–1007. doi: 10.1097/j.pain.0000000000000160, PMID: 25844555 PMC4450869

[ref60] Vaegter H. B. Støten M. Silseth S. L. Erlangsen A. Handberg G. Sondergaard S. . (2019). Cause-specific mortality of patients with severe chronic pain referred to a multidisciplinary pain clinic: a cohort register-linkage study. Scand J Pain 19, 93–99. doi: 10.1515/sjpain-2018-0094, PMID: 30205653

[ref61] Van Tilburg M. A. Spence N. J. Whitehead W. E. Bangdiwala S. Goldston D. B. (2011). Chronic pain in adolescents is associated with suicidal thoughts and behaviors. J. Pain 12, 1032–1039. doi: 10.1016/j.jpain.2011.03.004, PMID: 21684217 PMC3178682

[ref62] Vickers A. J. Vertosick E. A. Lewith G. Macpherson H. Foster N. E. Sherman K. J. . (2018). Acupuncture for chronic pain: update of an individual patient data Meta-analysis. J. Pain 19, 455–474. doi: 10.1016/j.jpain.2017.11.005, PMID: 29198932 PMC5927830

[ref63] Von Elm E. Altman D. G. Egger M. Pocock S. J. Gøtzsche P. C. Vandenbroucke J. P. (2007). The strengthening the reporting of observational studies in epidemiology (STROBE) statement: guidelines for reporting observational studies. Lancet 370, 1453–1457. doi: 10.1016/S0140-6736(07)61602-X 18064739

[ref64] Wang Y.-F. Yu C.-C. Kuan A. S. Chen S.-P. Wang S.-J. (2021). Association between suicidal risks and medication-overuse headache in chronic migraine: a cross-sectional study. J. Headache Pain 22, 1–8. doi: 10.1186/s10194-021-01248-0 33971819 PMC8112025

[ref65] Wilson K. G. Heenan A. Kowal J. Henderson P. R. Mcwilliams L. A. Castillo D. (2017). Testing the interpersonal theory of suicide in chronic pain. Clin. J. Pain 33, 699–706. doi: 10.1097/AJP.0000000000000451, PMID: 27768608

[ref66] Wilson K. G. Kowal J. Henderson P. R. Mcwilliams L. A. Péloquin K. (2013). Chronic pain and the interpersonal theory of suicide. Rehabil. Psychol. 58, 111–115. doi: 10.1037/a0031390, PMID: 23438008 PMC3998981

[ref67] Yan Y. Hou J. Li Q. Yu N. X. (2023). Suicide before and during the COVID-19 pandemic: a systematic review with Meta-analysis. Int. J. Environ. Res. Public Health 20:3346. doi: 10.3390/ijerph20043346, PMID: 36834037 PMC9960664

[ref68] You J. Li H. Xie D. Chen R. Chen M. (2021). Acupuncture for chronic pain-related depression: a systematic review and Meta-analysis. Pain Res. Manag. 2021, 1–10. doi: 10.1155/2021/6617075 PMC7925064 33680223

