# Supplementary material for: Prevalence of suicidal behavior in patients with chronic pain: a systematic review and meta-analysis of observational studies
Source: Front Psychol. 2023 Sep 29;14:1217299. doi: 10.3389/fpsyg.2023.1217299 (PMC10576560; doi:10.3389/fpsyg.2023.1217299)
Supplement: Supplementary file 2 [file Table_2.docx]

**Supplementary File 2. Search strategies and results**

**Medline via PubMed**

|  | Searches | Results |
| --- | --- | --- |
| #1 | Suicide[MH] OR “Self Mutilation”[MH] OR self-harm OR self-poisoning OR self-injur* OR “Self Mutilation” OR “attempted suicide” OR suicid* | 127,803 |
| #2 | “Chronic Pain”[MH] OR “chronic pain” | 55,899 |
| #3 | “Cohort Studies”[MH] OR “Cross-sectional Studies”[MH] OR “Epidemiologic Studies”[MH] OR “cohort design”[TIAB] OR “cohort stud*”[TIAB] OR “cross-sectional analysis”[TIAB] OR “cross-sectional design”[TIAB] OR “cross-sectional stud*”[TIAB] OR “epidemiologic stud*”[TIAB] OR incidence[TIAB] OR “longitudinal design”[TIAB] OR “longitudinal stud*”[TIAB] OR “observational stud*”[TIAB] OR “population stud*”[TIAB] OR prevalence[TIAB] | 4,278,453 |
| #4 | #1 AND #2 AND #3 | **190** |

**EMBASE via Elsevier**

|  | Searches | Results |
| --- | --- | --- |
| #1 | ‘suicidal behavior’/exp OR ‘suicide’/exp OR ‘automutilation’/exp OR self-harm OR self-poisoning OR self-injur* OR “Self Mutilation” OR “attempted suicide” OR suicid* | 174,841 |
| #2 | ‘chronic pain’/exp OR ‘chronic pain’ | 97,061 |
| #3 | 'cross-sectional study'/exp OR 'cohort design':ab,ti OR 'cohort stud*':ab,ti OR 'cross-sectional analysis':ab,ti OR 'cross-sectional design':ab,ti OR 'cross-sectional stud*':ab,ti OR 'epidemiologic stud*':ab,ti OR incidence:ab,ti OR 'longitudinal design':ab,ti OR 'longitudinal stud*':ab,ti OR 'observational stud*':ab,ti OR 'population stud*':ab,ti OR prevalence:ab,ti | 3,221,951 |
| #4 | #1 AND #2 AND #3 | **282** |

**Cochrane Library**

|  | Searches | Results |
| --- | --- | --- |
| #1 | MH descriptor: [Suicide] explode all trees | 1,509 |
| #2 | MH descriptor: [Self Mutilation] explode all trees | 37 |
| #3 | self-harm OR self-poisoning OR self-injur* OR “Self Mutilation” OR “attempted suicide” OR suicid* | 8,775 |
| #4 | MeSH descriptor: [Chronic Pain] explode all trees | 3,178 |
| #5 | chronic pain | 35,821 |
| #6 | (#1 OR #2 OR #3) AND (#4 OR #5) in Trials | **169** |

**CINAHL via EBSCO**

|  | Searches | Results |
| --- | --- | --- |
| #1 | SU Suicide OR SU Self Mutilation OR TX self-harm OR TX self-poisoning OR TX self-injur* OR TX Self Mutilation OR TX attempted suicide OR TX suicid* | 92838 |
| #2 | SU Chronic Pain OR TX Chronic pain | 147,980 |
| #3 | SU Cohort Studies OR SU Cross-sectional Studies OR SU Epidemiologic Studies OR TI cohort design OR AB cohort design OR TI cohort stud* OR AB cohort stud* OR TI cross-sectional analysis OR AB cross-sectional analysis OR TI cross-sectional design OR AB cross-sectional design OR TI cross-sectional stud* OR AB cross-sectional stud* OR TI epidemiologic stud* OR AB epidemiologic stud* OR TI incidence OR AB incidence OR TI longitudinal design OR AB longitudinal design OR TI longitudinal stud* OR AB longitudinal stud* OR TI observational stud* OR AB observational stud* OR TI population stud* OR AB population stud* OR TI prevalence OR AB prevalence | 1,081,202 |
| #4 | #1 AND #2 AND #3 | **1,410** |

**PsycARTICLES via ProQuest**

|  | Searches | Results |
| --- | --- | --- |
| #1 | SU(Suicide) OR SU(“Self Mutilation”) OR self-harm OR self-poisoning OR self-injur* OR “Self Mutilation” OR “attempted suicide” OR suicid* | 20,256 |
| #2 | SU(Chronic Pain) OR “chronic pain” | 3,452 |
| #3 | SU(Cohort Studies) OR SU(Cross-sectional Studies) OR SU(Epidemiologic Studies) OR TI(cohort design) OR AB(cohort design) OR TI(cohort stud*) OR AB(cohort stud*) OR TI(cross-sectional analysis) OR AB(cross-sectional analysis) OR TI(cross-sectional design) OR AB(cross-sectional design) OR TI(cross-sectional stud*) OR AB(cross-sectional stud*) OR TI(epidemiologic stud*) OR AB(epidemiologic stud*) OR TI(incidence) OR AB(incidence) OR TI(longitudinal design) OR AB(longitudinal design) OR TI(longitudinal stud*) OR AB(longitudinal stud*) OR TI(observational stud*) OR AB(observational stud*) OR TI(population stud*) OR AB(population stud*) OR TI(prevalence) OR AB(prevalence) | 18,934 |
| #4 | #1 AND #2 | **594** |
