# Supplementary material for: Prevalence of suicidal behavior in patients with chronic pain: a systematic review and meta-analysis of observational studies
Source: Front Psychol. 2023 Sep 29;14:1217299. doi: 10.3389/fpsyg.2023.1217299 (PMC10576560; doi:10.3389/fpsyg.2023.1217299)
Supplement: Supplementary file 3 [file Table_3.docx]

**Supplementary File 3. Documents excluded after full text review**

**[1] conference abstract (n = 22)**

1. Cheng Y, Marmaduke D, Crucitti A, Hoog S, Strombom I, Hornbuckle K. The risk of suicidal behavior among patients with diabetic peripheral neuropathic pain, low back pain, fibromyalgia, or osteoarthritis. Pharmacoepidemiology and Drug Safety (PDS). 2009;18(S1):S48-S9.

2. Bruns D, Fishbain D, Disorbio J, Lewis J. Predictors of homicide-suicide ideation in acute and chronic pain patients. Journal of Pain. 2010;11(4):S12.

3. Fishbain DA, Bruns D, Meyer LJ, Lewis JE, Gao J, Disorbio JM. Is endorsement of preference for death over disability associated with suicidality in chronic pain patients? Pain Medicine. 2011;12(3):495-6.

4. Iorio C, Pawluk E, Vermani M, Katzman MA. The prevalence of psychiatric features within a chronic pain population. Pain Research and Management. 2011;15(2):95.

5. Martinez RO, Santiago OJ, Espino D. Comparison of attitudes toward suicide and physician-assisted suicide in chronic pain scenarios between elderly whites and hispanics. Journal of the American Geriatrics Society. 2011;59:S144.

6. Patorno E, Hernández-Díaz S, Glynn RJ, Avorn J, Mogun H, Schneeweiss S. Risk of suicidal acts in new anticonvulsant or antidepressant drug users with chronic pain conditions. Pharmacoepidemiology and Drug Safety. 2012;21:444.

7. Cheatle M, Wasser T, Olugbodi A, Foster C. The prevalence and mediators of suicidal ideation in patients with chronic con-cancer pain. Journal of Pain. 2013;14(4):S24.

8. Stenager EN, Christiansen E, Handberg G, Jensen B. Suicide attempts in chronic pain patients. A register study. European Psychiatry. 2013;28.

9. Noyman-Veksler G, Lerman SF, Joiner TE, Brill S, Rudich Z, Shalev H, et al. Associations between psychosocial self-concept and coping variables and suicide ideation in chronic pain patients. Psychosomatic Medicine. 2015;77(3):A47.

10. Marini S, Lalli V, Sergi MR, Costantini A, Gambi F, Di Giannantonio M. Suicidal risk in chronic pain patients under intrathecal ziconotide treatment: An observational study. European Neuropsychopharmacology. 2016;26:S477-S8.

11. Romero Guillena SL, Florido Puerto AI, De Ingunza Barreiro E, Asencio Cabrera VD, Guillen Benitez M. Pain as a precursor of suicidal behaviours. European Neuropsychopharmacology. 2016;26:S388.

12. Tripp D, Nickel JC, Krsmanovic A, Pontari M, Moldwin R, Mayer R, et al. Depression and catastrophizing predicts suicidal ideation in tertiary care patients with interstitial cystitis/bladder pain syndrome (IC/BPS). Journal of Urology. 2016;195(4):e956.

13. Almeida RR, Fintelmann FJ, Mansouri MR, Marchiori E, Lev MH, Flores EJ. Can short term pain control result in long term suffering? 15-yr analysis of opioid prescription and medical history of patients presenting with thoracic complications from IV drug abuse. Journal of Thoracic Imaging. 2017;32(4):W33-W4.

14. Lovejoy T, Demidenko M, Morasco B, Meath T, Dobscha S. Suicidal ideation and behaviors following clinician-initiated prescription opioid discontinuation among long-term opioid users. Journal of Pain. 2017;18(4):S36.

15. Mancini V, Marini S, Lalli V, Sergi MR, Gambi F, Costantini A, et al. Suicide ideation, anger and impulsiveness in chronic pain patients before and after pain relief: An observational study. European Neuropsychopharmacology. 2017;27:S875-S6.

16. Pergolizzi J, Raffa R, Taylor R, Lequang JA, Colucci D, Passik S. The risk of suicide risk in chronic pain patients. Postgraduate Medicine. 2017;129(SUPPL 1):34-5.

17. Tripp D, Nickel JC, Dueck J, Muere A, Yurgan H, Gierc M. Biopsychosocial predictors of suicidality in patients with interstitial cystitis/bladder pain syndrome. Journal of Urology. 2017;197(4):e386.

18. Legarreta M, Sheth C, Rogowska J, McGlade E, Yurgelun-Todd D. Orbitofrontal functional connectivity and perceived pain disability in veterans with suicide ideation and suicide attempts. Neuropsychopharmacology. 2019;44:292-3.

19. Rojas AM, Flynn HA, Worts PR, Chandler GS. Rates and co-occurrences of psychological risk factors among chronic pain patients. Pain Physician. 2019;22(4):E391.

20. Androulakis XM, Sico JJ, Giakas A, Reyes D. Suicide attempt incidence and psychiatric comorbidities in veterans with chronic head, neck and back pain from 2001 to 2010: Trends and associations. Headache. 2021;61(SUPPL 1):43.

21. Delcoigne B, Horne A, Omarsdottir S, Reutfors J, Askling J. Psychiatric disorders in juvenile idiopathic arthritis-a population-based cohort study. Annals of the Rheumatic Diseases. 2021;80(SUPPL 1):939.

22. Gu X, Chen TC, Steinke D, Chen LC. Investigating the risk of severe adverse events in gabapentinoid users with chronic non-cancer pain in English primary care. Pharmacoepidemiology and Drug Safety. 2022;31:3.

**[2] duplicate (n = 1)**

1. Fisher BJ, Haythornthwaite JA, Heinberg LJ, Clark M, Reed J. Suicidal intent in patients with chronic pain. Revista de la Sociedad Espanola del Dolor. 2002;9(3):146-56.

**[3] review article (n = 2)**

1. Courtet P, Olie E. Social pain at the core of suicidal behavior. Encephale. 2019;45:S7‐S12.

2. Gill H, Perez CD, Gill B, El-Halabi S, Lee Y, Lipsitz O, et al. The Prevalence of Suicidal Behaviour in Fibromyalgia Patients. Progress in neuro-psychopharmacology & biological psychiatry. 2021;108:110078.

**[4] studies including conditions other than pain (n = 12)**

1. van Tilburg MA, Spence NJ, Whitehead WE, Bangdiwala S, Goldston DB. Chronic pain in adolescents is associated with suicidal thoughts and behaviors. The journal of pain. 2011;12(10):1032-9.

2. Sansone RA, Watts DA, Wiederman MW. Pain, Pain Catastrophizing, and History of Intentional Overdoses and Attempted Suicide. Pain Practice. 2014;14(2):E29-32.

3. Ilgen MA, Bohnert ASB, Ganoczy D, Bair MJ, McCarthy JF, Blow FC. Opioid dose and risk of suicide. Pain. 2016;157(5):1079-84.

4. Coplan PM, Sessler NE, Harikrishnan V, Singh R, Perkel C. Comparison of abuse, suspected suicidal intent, and fatalities related to the 7-day buprenorphine transdermal patch versus other opioid analgesics in the National Poison Data System. Postgrad Med. 2017;129(1):55-61.

5. Petrosky E, Harpaz R, Fowler KA, Bohm MK, Helmick CG, Yuan K, et al. Chronic Pain Among Suicide Decedents, 2003 to 2014: Findings From the National Violent Death Reporting System. Ann Intern Med. 2018;169(7):448-55.

6. Owen-Smith AA, Ahmedani BK, Peterson E, Simon GE, Rossom RC, Lynch FL, et al. The Mediating Effect of Sleep Disturbance on the Relationship Between Nonmalignant Chronic Pain and Suicide Death. Pain practice : the official journal of World Institute of Pain. 2019;19(4):382-9.

7. Sommer JL, Blaney C, El-Gabalawy R. A population-based examination of suicidality in comorbid generalized anxiety disorder and chronic pain. J Affect Disord. 2019;257:562-7.

8. Agnoli A, Xing G, Tancredi DJ, Magnan E, Jerant A, Fenton JJ. Association of Dose Tapering With Overdose or Mental Health Crisis Among Patients Prescribed Long-term Opioids. Jama. 2021;326(5):411-9.

9. Roughan WH, Campos AI, García-Marín LM, Cuéllar-Partida G, Lupton MK, Hickie IB, et al. Comorbid Chronic Pain and Depression: Shared Risk Factors and Differential Antidepressant Effectiveness. Front Psychiatry. 2021;12:643609.

10. Wright N, Ramirez MR. A cross sectional study of non-medical use of prescription opioids and suicidal behaviors among adolescents. Injury Epidemiology. 2021;8(1):1-7.

11. Hilgeman MM, Cramer DRJ, Hoch MC, Collins AN, Zabelski S, Heebner NR. A Pilot Study Comparing Two Measures of Perceived Health Services Access Among Military Veterans With Musculoskeletal Injuries and Mental Health Conditions. Military medicine. 2022.

12. Shor R, Borowski S, Zelkowitz RL, Pineles SL, Copeland LA, Finley EP, et al. The transition to civilian life: Impact of comorbid PTSD, chronic pain, and sleep disturbance on veterans’ social functioning and suicidal ideation. Psychological Trauma: Theory, Research, Practice, and Policy. 2022.

**[5] studies where it is not clear whether the participants' pain is chronic (n = 18)**

1. Kikuchi N, Ohmori-Matsuda K, Shimazu T, Sone T, Kakizaki M, Nakaya N, et al. Pain and risk of completed suicide in Japanese men: a population-based cohort study in Japan (ohsaki cohort study). Journal of Pain & Symptom Management. 2009;37(3):316-24.

2. Calandre EP, Vilchez JS, Molina-Barea R, Tovar MI, Garcia-Leiva JM, Hidalgo J, et al. Suicide attempts and risk of suicide in patients with fibromyalgia: A survey in Spanish patients. Rheumatology. 2011;50(10):1889-93.

3. Fitzcharles MA, Ste-Marie PA, Gamsa A, Ware MA, Shir Y. Opioid use, misuse, and abuse in patients labeled as fibromyalgia. The American journal of medicine. 2011;124(10):955-60.

4. Jimenez-Rodríguez I, Garcia-Leiva JM, Jimenez-Rodriguez BM, Condés-Moreno E, Rico-Villademoros F, Calandre EP. Suicidal ideation and the risk of suicide in patients with fibromyalgia: a comparison with non-pain controls and patients suffering from low-back pain. Neuropsychiatr Dis Treat. 2014;10:625-30.

5. Calandre EP, Navajas-Rojas MA, Ballesteros J, Garcia-Carrillo J, Garcia-Leiva JM, Rico-Villademoros F. Suicidal ideation in patients with fibromyalgia: a cross-sectional study. Pain practice : the official journal of World Institute of Pain. 2015;15(2):168-74.

6. Koenig J, Oelkers-Ax R, Parzer P, Haffner J, Brunner R, Resch F, et al. The association of self-injurious behaviour and suicide attempts with recurrent idiopathic pain in adolescents: evidence from a population-based study. Child Adolesc Psychiatry Ment Health. 2015;9:32.

7. Triñanes Y, González-Villar A, Gómez-Perretta C, Carrillo-de-la-Peña MT. Suicidality in chronic pain: predictors of suicidal ideation in fibromyalgia. Pain practice : the official journal of World Institute of Pain. 2015;15(4):323-32.

8. Yang S-E, Park Y-G, Han K, Min J-A, Kim S-Y. Dental pain related to quality of life and mental health in South Korean adults. Psychology, Health & Medicine. 2016;21(8):981-92.

9. Lin S-L, Wu S-L, Huang H-T, Lung F-W, Chi T-C, Yang J-W. Can a 10-Minute Questionnaire Identify Significant Psychological Issues in Patients With Temporomandibular Joint Disease? Journal of Oral & Maxillofacial Surgery (02782391). 2017;75(9):1856-65.

10. Jung JH, Seok H, Kim JH, Song GG, Choi SJ. Association between osteoarthritis and mental health in a Korean population: a nationwide study. Int J Rheum Dis. 2018;21(3):611-9.

11. Stefko-Comte L, Guérin J, Colin F, Perrot S, Coste J, Laroche F. Psychological comorbidities and fibromyalgia - Evaluation of a new dimension: Perceived injustice. About an online survey of 4516 patients. Douleur et Analgesie. 2018;31(4):217-22.

12. Wolfe F, Ablin J, Baker JF, Diab R, Guymer EK, Littlejohn GO, et al. All-cause and cause-specific mortality in persons with fibromyalgia and widespread pain: An observational study in 35,248 persons with rheumatoid arthritis, non-inflammatory rheumatic disorders and clinical fibromyalgia. Seminars in Arthritis & Rheumatism. 2020;50(6):1457-64.

13. Ashrafioun L, Bishop TM, Pigeon WR. The Relationship Between Pain Severity, Insomnia, and Suicide Attempts Among a National Veteran Sample Initiating Pain Care. Psychosom Med. 2021;83(7):733-8.

14. Gmuca S, Sonagra M, Xiao R, Miller KS, Thomas NH, Young JF, et al. Suicidal risk and resilience in juvenile fibromyalgia syndrome: a cross-sectional cohort study. Pediatr Rheumatol Online J. 2021;19(1):3.

15. Liu X, Liu ZZ, Yang Y, Jia CX. Prospective Associations of Frequent Pain Symptoms With Suicidal Behavior in Adolescents. The journal of pain. 2021;22(7):852-63.

16. Roy R, Sommer JL, Bolton JM, El-Gabalawy R. Understanding correlates of suicidality among those with usual pain and discomfort: A Canadian nationally representative study. J Psychosom Res. 2021;151:110651.

17. Baeza-Velasco C, Hamonet C, Montalescot L, Courtet P. Suicidal Behaviors in Women With the Hypermobile Ehlers-Danlos Syndrome. Arch Suicide Res. 2022;26(3):1314-26.

18. Luo W, Cao X, Zhao J, Yang J, Cen Y, He J, et al. Health-related quality of life and associated factors in Chinese menstrual migraine patients: a cross-sectional study. BMC Women's Health. 2022;22(1):1-7.

**[6] studies that included both chronic and acute pain (n = 7)**

1. Rozen TD, Fishman RS. Cluster Headache in the United States of America: Demographics, Clinical Characteristics, Triggers, Suicidality, and Personal Burden. Headache: The Journal of Head & Face Pain. 2012;52(1):99-113.

2. Fuller-Thomson E, Ramzan N, Baird SL. Arthritis and suicide attempts: findings from a large nationally representative Canadian survey. Rheumatology international. 2016;36(9):1237-48.

3. Choong CK, Ford JH, Nyhuis AW, Joshi SG, Robinson RL, Aurora SK, et al. Clinical Characteristics and Treatment Patterns Among Patients Diagnosed With Cluster Headache in U.S. Healthcare Claims Data. Headache: The Journal of Head & Face Pain. 2017;57(9):1359-74.

4. Friedman LE, Zhong QY, Gelaye B, Williams MA, Peterlin BL. Association Between Migraine and Suicidal Behaviors: A Nationwide Study in the USA. Headache: The Journal of Head & Face Pain. 2018;58(3):371-80.

5. Rozen TD. Cluster Headache Clinical Phenotypes: Tobacco Nonexposed (Never Smoker and No Parental Secondary Smoke Exposure as a Child) versus Tobacco‐Exposed: Results from the United States Cluster Headache Survey. Headache: The Journal of Head & Face Pain. 2018;58(5):688-99.

6. Konietzny K, Chehadi O, Levenig C, Kellmann M, Kleinert J, Mierswa T, et al. Depression and suicidal ideation in high-performance athletes suffering from low back pain: The role of stress and pain-related thought suppression. Eur J Pain. 2019;23(6):1196-208.

7. Fuller-Thomson E, Hodgins GA. Suicide Attempts among Those with Migraine: Findings from a Nationally Representative Canadian Study. Arch Suicide Res. 2020;24(sup1):360-79.

**[7] studies that did not report any outcome related to suicidal behavior (n = 1)**

1. Men F, Fischer B, Urquia ML, Tarasuk V. Food insecurity, chronic pain, and use of prescription opioids. SSM Popul Health. 2021;14:100768.

**[8] studies that did not report the prevalence of suicidal behavior (n = 13)**

1. Novy DM, Nelson DV, Berry LA, Averill PM. What does the Beck Depression Inventory measure in chronic pain?: a reappraisal. Pain. 1995;61(2):261-70.

2. Fisher BJ, Haythornthwaite JA, Heinberg LJ, Clark M, Reed J. Suicidal intent in patients with chronic pain. Pain. 2001;89(2-3):199-206.

3. Manchikanti KN, Manchikanti L, Damron KS, Pampati V, Fellows B. Increasing deaths from opioid analgesics in the United States: An evaluation in an interventional pain management practice. Journal of Opioid Management. 2008;4(5):271-83.

4. Gilbert JW, Wheeler GR, Storey BB, Mick G, Richardson G, Westerfield G, et al. Suicidality in chronic noncancer pain patients. The International journal of neuroscience. 2009;119(10):1968-79.

5. Tang NK, Salkovskis PM, Hodges A, Soong E, Hanna MH, Hester J. Chronic pain syndrome associated with health anxiety: a qualitative thematic comparison between pain patients with high and low health anxiety. The British journal of clinical psychology. 2009;48(Pt 1):1-20.

6. Kowal J, Wilson KG, McWilliams LA, Péloquin K, Duong D. Self-perceived burden in chronic pain: relevance, prevalence, and predictors. Pain. 2012;153(8):1735-41.

7. Margari F, Lorusso M, Matera E, Pastore A, Zagaria G, Bruno F, et al. Aggression, impulsivity, and suicide risk in benign chronic pain patients - a cross-sectional study. Neuropsychiatr Dis Treat. 2014;10:1613-20.

8. Bryan CJ, Kanzler KE, Grieser E, Martinez A, Allison S, McGeary D. A Shortened Version of the Suicide Cognitions Scale for Identifying Chronic Pain Patients at Risk for Suicide. Pain Practice. 2017;17(3):371-81.

9. Brown LA, Lynch KG, Cheatle M. Pain catastrophizing as a predictor of suicidal ideation in chronic pain patients with an opiate prescription. Psychiatry Res. 2020;286:112893.

10. Orr MF, Rogers AH, Shepherd JM, Buckner JD, Ditre JW, Bakhshaie J, et al. Is there a relationship between cannabis use problems, emotion dysregulation, and mental health problems among adults with chronic pain? Psychology, Health & Medicine. 2020;25(6):742-55.

11. Bisson EJ, Sawhney M, Duggan S, Good MA, Wilson R. Pain, mental health, and health care utilization of military Veterans compared to civilians in a chronic pain clinic. Journal of Military, Veteran & Family Health. 2021;7:20-8.

12. Chytas V, Costanza A, Mazzola V, Luthy C, Galani V, Bondolfi G, et al. Possible Contribution of Meaning in Life in Patients With Chronic Pain and Suicidal Ideation: Observational Study. JMIR formative research. 2022;6(6):e35194.

13. Meerwijk EL, Adams RS, Larson MJ, Highland KB, Harris AHS. Dose of Exercise Therapy Among Active Duty Soldiers With Chronic Pain Is Associated With Lower Risk of Long-Term Adverse Outcomes After Linking to the Veterans Health Administration. Mil Med. 2022.

**[9] studies that reported the prevalence, but only presented it as a percentage, and the value was unclear (n = 8)**

1. Magni G, Rigatti-Luchini S, Fracca F, Merskey H. Suicidality in chronic abdominal pain: An analysis of the Hispanic Health and Nutrition Examination Survey (HHANES). Pain. 1998;76(1-2):137-44.

2. Balousek S, Plane MB, Fleming M. Prevalence of interpersonal abuse in primary care patients prescribed opioids for chronic pain. J Gen Intern Med. 2007;22(9):1268-73.

3. Ilgen MA, Zivin K, McCammon RJ, Valenstein M. Pain and suicidal thoughts, plans and attempts in the United States. Gen Hosp Psychiatry. 2008;30(6):521-7.

4. Ekholm O, Kurita GP, Hjsted J, Juel K, Sjgren P. Chronic pain, opioid prescriptions, and mortality in Denmark: A population-based cohort study. Pain. 2014;155(12):2486-90.

5. Stenager E, Christiansen E, Handberg G, Jensen B. Suicide attempts in chronic pain patients. A register-based study. Scand J Pain. 2014;5(1):4-7.

6. Campbell G, Darke S, Degenhardt L, Townsend H, Carter G, Draper B, et al. Prevalence and Characteristics Associated with Chronic Noncancer Pain in Suicide Decedents: A National Study. Suicide Life Threat Behav. 2020;50(4):778-91.

7. Grocott B, Sommer JL, El-Gabalawy R. Usual presence and intensity of pain are differentially associated with suicidality across chronic pain conditions: A population-based study. J Psychosom Res. 2021;148:110557.

8. Jolly T, Vadukapuram R, Trivedi C, Mansuri Z, Adnan M, Cohen SP, et al. Risk of Suicide in Patients With Major Depressive Disorder and Comorbid Chronic Pain Disorder: An Insight From National Inpatient Sample Data. Pain Physician. 2022;25(6):419-25.

**[10] a study reporting mixed prevalence of suicide and homicide (n = 1)**

1. Fishbain DA, Bruns D, Lewis JE, Disorbio JM, Gao J, Meyer LJ. Predictors of homicide-suicide affirmation in acute and chronic pain patients. Pain Med. 2011;12(1):127-37.

**[11] a study of participants who already had chronic pain and suicidal ideation (n = 1)**

1. Fuller-Thomson E, Kotchapaw LD. Remission From Suicidal Ideation Among Those in Chronic Pain: What Factors Are Associated With Resilience? The journal of pain. 2019;20(9):1048-56.
