# Supplementary material for: Prevalence of suicidal behavior in patients with chronic pain: a systematic review and meta-analysis of observational studies
Source: Front Psychol. 2023 Sep 29;14:1217299. doi: 10.3389/fpsyg.2023.1217299 (PMC10576560; doi:10.3389/fpsyg.2023.1217299)
Supplement: Supplementary file 4 [file Table_4.docx]

**Supplementary File 4. Factors explaining the association between chronic pain and suicidal behavior**

| **Study** | **Investigated factors investigated (from Logistic regression)** |
| --- | --- |
| **Smith 2004** | **1. with current passive suicidal ideation** [Type 2] 1) SR(+): abdominal pain, neuropathic pain, family history; 2) NSR: BDI (no item 9) **2. with current active suicidal ideation** [Type 2] 1) SR(+): neuropathic pain, family history; 2) NSR: abdominal pain, BDI (no item 9) |
| **Ratcliffe 2008** | **1. with past year suicidal ideation** [Type 1] 1) SR(+): migraine, arthritis, back problems, fibromyalgia, other chronic pain / [Type 2] 1) SR(+): migraine, back problems, other chronic pain; 2) NSR: arthritis, fibromyalgia **2. with past year suicide attempts** [Type 1] 1) SR(+): migraine, arthritis, back problems, fibromyalgia, other chronic pain / [Type 2] 1) SR(+): migraine, back problems, other chronic pain; 2) NSR: arthritis, fibromyalgia |
| **Poole 2009** | NA |
| **Kanzler 2012** | **1. with current suicide ideation** [Type 2] 1) SR(+): burdensomeness; 2) NSR: age, gender, race, marital status, depression, pain severity |
| **Dutta 2013** | NA |
| **Cheatle 2014** | **1. with current suicide ideation** [Type 1] 1) SR(+): history of sexual/physical abuse, family history of depression, socially withdrawn; 2) NSR: pain location - abdominal, pain location - extremity, pain location - generalized, sleep disorder, work/school disruption, family discord, pre-pain depression, pre-pain history of suicide ideation, history of suicide attempts, family history of suicide attempts |
| **Campbell 2015a** | NA *The authors analyzed the association between bipolar disorder and suicidality, but not the association between chronic pain condition and suicidality. |
| **Campbell 2015b** | **1. with past year suicidal behaviors** [Type 1] 1) SR(+): past 12 month depression, past 12 month generalized anxiety disorder, past 12 month PTSD, past 12 month alcohol use disorder, subjective physical health fair-to-poor; 2) NSR: sex, education, employed, past 12 month drug use disorder; 3) SR(-): age over 60 years, married/de facto |
| **Ciaramella 2015** | NA *The authors analyzed the association between chronic pain condition and psychiatric diseases, but not the association between chronic pain condition and suicidality. |
| **Im 2015** | NA *The authors analyzed the association between opioid prescription and suicide attempts, but not the association between chronic pain condition and suicidality. |
| **Bertoli 2016** | NA |
| **Campbell 2016** | **1. with past year suicidal ideation** [Type 2] 1) SR(+): lifetime depression, lifetime suicide attempt; 2) NSR: age, sex, marriage, unemployed, income, 12 month anxiety, 12 month PTSD, borderline personality disorder, childhood abuse, social support, headache, fibromyalgia, 12 month number of pain conditions, pain severity, pain interference, pain coping and self-efficacy, sleep problem, short form 12 physical health, oral morphine equivalent, current benzodiazepine **2. with past year suicide attempt** [Type 2] 1) SR(-): pain coping and self-efficacy; 2) NSR: age, sex, 12 month anxiety, 12 month PTSD, short form 12 physical health |
| **Bromberg 2017** | **1. with past 2 weeks suicide ideation** [Type 2] 1) SR(+): depressive symptoms; 2) NSR: sex, race, family income, loneliness, family functioning, pain intensity, pain bother, functional disability; 3) SR(-): self-worth |
| **Ciaramella 2017** | NA |
| **Wilson 2017** | **1. with current suicide ideation** [Type 1] 1) SR(+): previous suicide attempts, hopelessness, perceived burdensomeness; 2) NSR: depression, insomnia, pain intensity, pain catastrophizing, functional limitations, pain self-efficacy, thwarted belongingness; 3) SR(-): sex (male) |
| **Blakey 2018** | **1. with current suicide ideation** [Type 1] 1) SR(+): race (White), pain interference; 2) NSR: age, gender, pain intensity / [Type 2] 1) SR(+): race (White), PTSD, alcohol abuse, major depressive disorder; 2) NSR: age, gender, pain intensity, pain interference, TBI, TBI+PTSD, drug abuse |
| **Lewcun 2018** | **1. with past 2 weeks passive or active suicide ideation** [Type 2] 1) SR(+): depressive symptoms; 2) NSR: pain duration |
| **Vaegter 2019** | NA |
| **Abdelghani 2020** | **1. with lifetime suicidal ideation** [Type 1] SR(+): drug use disorder / [Type 2] SR(+): drug use disorder **2. with lifetime suicide attempts** [Type 1] SR(+): drug use disorder / [Type 2] NSR: drug use disorder |
| **Androulakis 2021** | **1. with Suicidal attempt** [Type 2] 1) SR(+): sex (male), race (Asian), chronic headache, traumatic brain injury: adjustment disorder, antisocial personality, anxiety+headache, ADHD, bipolar disorder, borderline personality disorder, delusional disorder, dependent personality, depression, narcissistic personality, OCD, PTSD, schizoaffective disorder, schizophrenia; 2) SR(-): age at diagnosis, race (Black), race (unknown), ethnicity (Hispanic/Latino), ethnicity (unknown), anxiety-headache |
| **Rojas 2021** | NA |
| **Wang 2021** | **1. with lifetime suicidal ideation** [Type 1] SR(+): medication overuse headache / [Type 2] SR(+): medication overuse headache **2. with lifetime suicide attempt** [Type 1] SR(+): medication overuse headache / [Type 2] SR(+): medication overuse headache |
| **Song 2022** | **1. with current suicide ideation**  [Type 1] NSR: pain phenotype (low impact, moderate impact, high impact) **2. with suicide attempt**  [Type 1] NSR: pain phenotype (low impact, moderate impact, high impact) |

**Abbreviations.** ADHD, attention-deficit hyperactivity disorder; BDI, Beck Depression Inventory; NA, not applicable; NSR, not significantly related; OCD, obsessive-compulsive disorder; PTSD, posttraumatic stress disorder; SR(-), significantly related (negatively); SR(+), significantly related (positively); TBI, traumatic brain injury. **Note**. Type 1: adjusted for demographic and social factors; Type 2: adjusted for mental conditions including depression (If there are multiple similar models, the one with the most adjustment)
